# Discovery of new MD2 inhibitor from chalcone derivatives with anti-inflammatory effects in LPS-induced acute lung injury

**DOI:** 10.1038/srep25130

**Published:** 2016-04-27

**Authors:** Yali Zhang, Jianzhang Wu, Shilong Ying, Gaozhi Chen, Beibei Wu, Tingting Xu, Zhiguo Liu, Xing Liu, Lehao Huang, Xiaoou Shan, Yuanrong Dai, Guang Liang

**Affiliations:** 1 Chemical Biology Research Center at School of Pharmaceutical Sciences, Wenzhou Medical University, Wenzhou, Zhejiang 325035, China; 2 The 2nd Affiliated Hospital, Wenzhou Medical University, Wenzhou, Zhejiang 325035, China

## Abstract

Acute lung injury (ALI) is a life-threatening acute inflammatory disease with limited options available for therapy. Myeloid differentiation protein 2, a co-receptor of TLR4, is absolutely required for TLR4 sense LPS, and represents an attractive target for treating severe inflammatory diseases. In this study, we designed and synthesized 31 chalcone derivatives that contain the moiety of (*E*)-4-phenylbut-3-en-2-one, which we consider the core structure of current MD2 inhibitors. We first evaluated the anti-inflammatory activities of these compounds in MPMs. For the most active compound **20**, we confirmed that it is a specific MD2 inhibitor through a series of biochemical experiments and elucidated that it binds to the hydrophobic pocket of MD2 via hydrogen bonds with Arg^90^ and Tyr^102^ residues. Compound **20** also blocked the LPS-induced activation of TLR4/MD2 -downstream pro-inflammatory MAPKs/NF-κB signaling pathways. In a rat model with ALI induced by intracheal LPS instillation, administration with compound **20** exhibited significant protective effect against ALI, accompanied by the inhibition of TLR4/MD2 complex formation in lung tissues. Taken together, the results of this study suggest the specific MD2 inhibitor from chalcone derivatives we identified is a potential candidate for treating acute inflammatory diseases.

Acute lung injury (ALI) is a life-threatening disease characterized by diffuse pulmonary interstitial and alveolar edema that leads to respiratory failure and death^1^. Despite extensive research, its mortality rate remains quite high (approximately 40%)^2^,^3^. Research efforts targeting ALI treatment have focused primarily on the innate immune system, and have typically conceptually viewed ALI as a syndrome of hyperinflammation^4^. Lipopolysaccharide (LPS), a known endotoxin, is a component of outer membranes of Gram-negative bacteria and is a potent inducer of local acute inflammation^5^. Excessive inflammatory response, called “inflammatory storm”, leads to acute injury of organs including the lungs^6^. There are currently no effective methods of treating these acute inflammatory diseases, and pharmacotherapy for ALI is extremely limited^7^. Targeting the inhibition of cytokines has, however, been identified as an important potential strategy for treating inflammatory diseases^8^,^9^.

Toll-like receptors (TLRs) are key pattern-recognition receptors that recognize invading pathogens and induce innate and adaptive immune responses^10^,^11^. TLR4, one of the most extensively researched of these receptors, is responsible for pro-inflammatory activation by LPS^12^,^13^. However, TLR4 alone is not sufficient for recognizing LPS. Myeloid differentiation 2 (MD2), a co-receptor of TLR4, is absolutely required to sense most of the LPS lipid chains which interact with the hydrophobic pocket of MD2^14^,^15^,^16^. Upon binding with LPS, a receptor multimer that consists of two copies of TLR4/MD2/LPS complex is formed, triggering downstream signal transduction (such as MAPK phosphorylation and NF-κB activation,) which up-regulate the expression of a series of inflammatory mediators^12^,^14^. Previous researchers have conducted extensive study on the function of MD2, and confirmed its essential role in the pathogenesis of sepsis^17^,^18^. MD2 knockout mice do not respond to *E. coli* LPS, and thus can be survived from endotoxic shock^19^. Due to its importance in mediating the interaction between TLR4 and LPS, MD2 has been explored as a therapeutic target for treating acute inflammatory diseases.

As of now, several natural products and synthetic chemicals have been reported as MD2 inhibitors. For example, chalcone derivatives, xanthohumol and JSH, have been shown to significantly inhibit the LPS-induced TLR4 pathway by antagonizing the binding sites between LPS and MD2^20^,^21^. Several natural products such as curcumin, caffeic acid phenethyl ester, and 1-dehydro-10-gingerdione have also been reported to attenuate the LPS-induced inflammatory response by inhibiting MD2^22^,^23^,^24^. Interestingly, the chemical structures of currently reported small-molecule MD2 inhibitors share the same skeleton of (*E*)-4-phenylbut-3-en-2-one, indicating that this moiety may play an important role in MD2 inhibition (Supplementary Fig. S1).

Chalcones, natural substances that are common in fruits, vegetables, spices, tea, and soy-based foodstuffs, have attracted considerable interest from researchers and developers in recent years due to their pharmacological activities, including antimicrobial, anticancer, antifungal, antioxidant, and anti-inflammatory properties^25^. Recently, a number of chalcones have been found to inhibit inflammatory cytokine release both *in vivo* and *in vitro* and several showed MD2-inhibitory properties, although their structure-activity relationship and clinical efficiency in the treatment of acute inflammatory diseases remain unclear^20^,^21^,^26^,^27^. In the present study, we synthesized 31 chalcone derivatives and evaluated their cytokine-inhibitory activity *in vitro*. Among them, compound **20** was identified as a new and selective MD2 inhibitor which exhibited anti-inflammatory activity both *in vitro* and in an animal ALI model. The results suggest that compound **20**, a new MD2 inhibitor, has significant potential to be developed as a candidate for treating acute inflammatory diseases.

## Results

### Synthesis and Anti-inflammatory activity evaluation of the synthesized 31 chalcone derivatives

To identify potent analogues with optimal pharmacological properties, we extended the molecular diversity of the chalcone skeleton and synthesized 31 chalcone derivatives (**1**–**31**). Chalcones were synthesized by traditional aldol condensation of substituted benzaldehyde with prepared acetophenones in ethanolic NaOH or HCl solution. The purity was determined by thin-layer chromatography (250 μ silica gel 60 F_254_ glass plates) and the products were characterized by ^1^H-NMR, ^13^C-NMR, HRMS, and ESI-MS. The synthetic profiles of the compounds and their chemical structures are illustrated in Supplementary Fig. S2. The synthetic yields, melting points, ^1^H NMR, ^13^C-NMR, HRMS, and ESI-MS results of these novel compounds are described in detail in the Supplementary Information. The original ^1^H NMR spectra of compounds 1–31 were shown in Supplementary Fig. S3. Before use in biological experiments, compounds were recrystallized from CHCl_3_/EtOH and HPLC was used to verify their purity (≥95.0%).

Next, we systematically evaluated the effects of the synthesized chalcone derivatives on inflammatory cytokine production. LPS was used to induce the production of inflammatory cytokines TNF-α and IL-6 in mouse peritoneal macrophages (MPMs) in the presence or absence of chalcone derivatives. Xanthohumol, a natural MD2 inhibitor with anti-inflammatory properties, was used as a positive comparison. The results of the anti-inflammatory evaluation of 31 chalcones (Supplementary Fig. S2) showed that the majority of the tested compounds inhibited the LPS-induced overexpression of TNF-α and IL-6. Compounds **20**, **27** and **29** exhibited higher inhibition (54.8–63.1% inhibition) than xanthohumol against TNF-α expression. In the case of IL-6, compounds **3**, **20**, **24**, **25**, **26**, **29** and **31** showed stronger activity than xanthohumol with inhibition ranging from 71.1% to 96.6%. Among these derivatives, compound **20** showed the strongest inhibitory effect on LPS-induced expression of both TNF-α and IL-6. We were unable to observe any obvious structure-activity relationship in the study.

### Dose-dependent anti-inflammatory effects of active compounds

**Figure 1:**
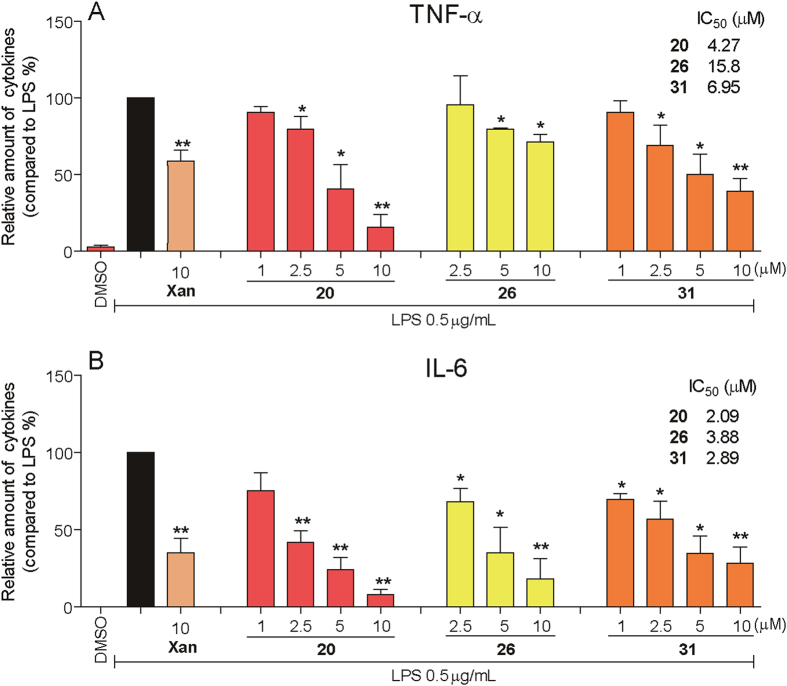
Inhibition of TNF-α and IL-6 production as indicated by chalcone derivatives. After plating and 24 h of growth, cells were pretreated for 2 h with the indicated concentrations of each chalcone derivative before stimulated with LPS (0.5 μg/mL) for 22 h. Culture medium and proteins were then collected. The TNF-α and IL-6 levels in the medium were determined by ELISA. Data are expressed as fold change relative to control values (samples treated with LPS alone), mean ± SEM. n ≥ 3. *p < 0.05 and **p < 0.01 vs. only-LPS stimulated group.

Dose-response experiments were conducted to obtain IC_50_ values for three active derivatives (**20**, **26** and **31**). MPMs were pretreated with compounds at a series of concentrations for 2 h, then incubated with LPS (0.5 μg/mL) for 22 h. As shown in Fig. 1, the three compounds demonstrated dose-dependent inhibitory effects against LPS-induced TNF-α and IL-6 release. The active compounds, even in 5 μM, exhibited more potent inhibitory activity than xanthohumol at 10 μM. Additionally, these three active compounds show no obvious cytotoxicity in macrophages (Supplementary Fig. S4). These results support our assertion that these active compounds are favorable the potential anti-inflammatory agents.

### Compound 20 as a selective MD2 inhibitor

**Figure 2:**
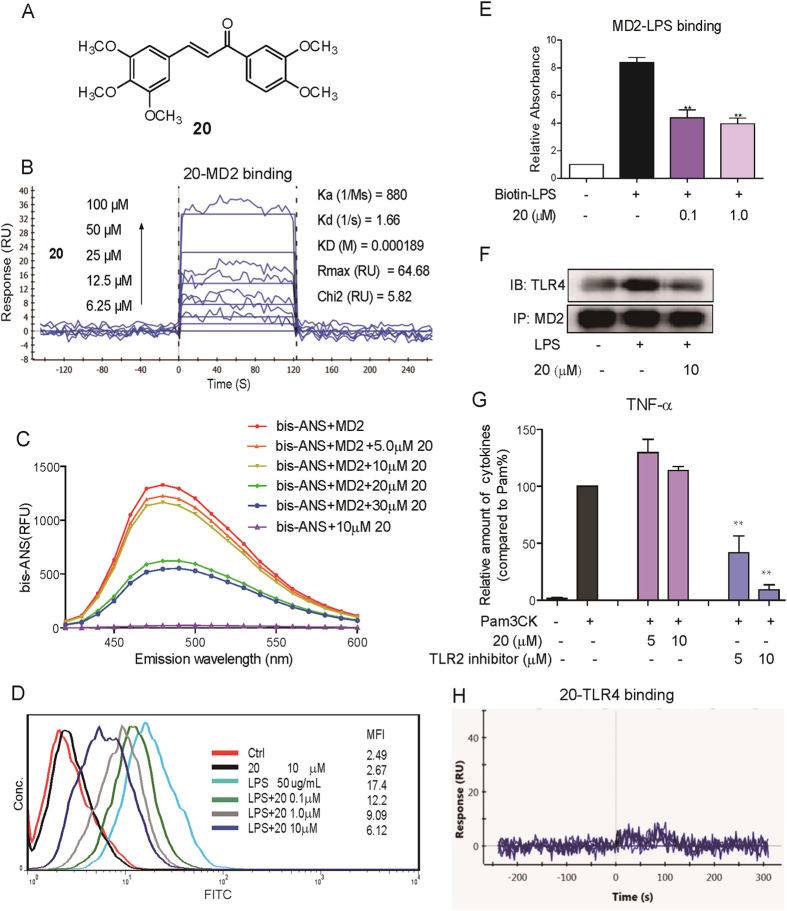
Compound 20 is a specific inhibitor of MD2. (**A**) Chemical structure of compound 20. (**B**) Surface plasmon resonance (SPR) analysis shows the direct interaction between compound **20** and rhMD2 protein. (**C**) Fluorescence measurements show that compound **20** inhibited bis-ANS binding to rhMD2 in a dose-dependent manner. (**D**) Flow cytometry analysis as used to detect the effects of compound **20** on FITC-LPS binding to cell surface. (**E**) Compound **20** displaces the biotin-LPS binding to rhMD2 determined by MD2 cell-free ELISA assay. (**F**) Immunoprecipitation assay shows that compound **20** pretreatment at 10 μM significantly reduced the formation of TLR4-MD2 complex induced by 0.5 μg/mL LPS. The gels were run under the same experimental conditions and were shown as cropped gels/blots (Cropped gels/blots with indicated cropping lines are shown in Supplementary Figure S8). (**G**) RAW264.7 macrophages after pretreatment with vehicle, compound **20** (5 or 10 μM), or TLR2 inhibitor (5 or 10 μM) for 30 min followed by incubation with Pam3CK (0.1 μg/mL) for 12 h. The protein level of TNF-α and IL-6 in the media was measured by ELISA and normalized to the respective total protein amount. (**H**) SPR analysis shows no direct interaction between compound **20** and rhTLR4 protein.

Compound **20** showed the strongest anti-inflammatory activity *in vitro*, so it was selected for subsequent target study. The chemical structure of **20** is shown in Fig. 2A. We tested the direct interaction between compound **20** and recombinant human MD2 (rhMD2) protein by Surface Plasmon Resonance (SPR) assay and fluorescence spectroscopy. SPR analysis showed that compound **20** exhibited recognizable binding to rhMD2 protein in a dose-dependent manner, with a KD value of 189 μM (Fig. 2B), while the KD value of xanthohumol binding to MD2 is 460 μM (Supplementary Fig. S5). Fluorescent probe bis-ANS was also used to study the lipid-binding sites of several proteins, including MD2^28^,^29^. As shown in Fig. 2C, the fluorescence produced by bis-ANS-rhMD2 binding was dose-dependently reduced by incubation with compound **20**, indicating a competitive binding of compound **20** toward the hydrophobic pocket of MD2. Because we observed compound **20** to bind directly to MD2, we next examined whether it interferes with interactions between LPS-MD2 and TLR4-MD2. Macrophages were incubated with FITC-conjugated LPS (FITC-LPS), then subjected to flow cytometry analysis. Pre-treatment with different doses of compound **20** dose-dependently reduced FITC-LPS binding to MD2 in cell surface membranes, with a 65% inhibition at 10 μM in terms of mean fluorescence intensity (Fig. 2D). To further confirm the antagonistic effect of **20** on LPS-MD2 interaction at the molecular level, we established a specific ELISA system. Treatment with **20** inhibited the biotin-labeled LPS (biotin-LPS) binding to rhMD2 in a dose-dependent manner (Fig. 2E). Upon binding with LPS, there was increased formation of TLR4/MD2 complex, triggering the downstream pro-inflammatory pathway. We determined the influence of **20** on the LPS-induced increase of TLR4/MD2 complex by immunoprecipitation. Compared to the vehicle, LPS alone largely increased the amount of TLR4/MD2 complex, while pretreatment with **20** inhibited the increase of TLR4/MD2 complex to the vehicle level (Fig. 2F).

To further examine whether compound **20** specifically inhibited LPS-induced TLR4 signaling, TLR2, another member of the TLR family associated with MD2-independent inner immune signaling, was also investigated^30^. Pam3CK is a specific agonist of the TLR2 signaling pathway. As shown in Fig. 2G, Pam3CK significantly increased the pro-inflammatory cytokine TNF-α level in MPMs, while pretreatment with **20** showed no inhibition against TNF-α release. This observation implies that compound **20** specifically targets the MD2 protein, and is unable to affect the MD2-independent TLR2 pro-inflammatory pathway. We also tested the interaction between compound **20** and TLR4; our SPR assay showed that compound **20** had no binding affinity to rhTLR4 protein (Fig. 2H). We then examined the direct effects of compound **20** on TLR4/ MD2-downstream kinases, such as ERK1/2, IRAK1, and IKKβ, which are involved in the TLR4 pro-inflammatory signaling pathway, using the Caliper Mobility Shift Assay. As expected, **20** showed no obvious inhibitory activity on these three kinases (Supplementary Fig. S6). These results altogether demonstrate that MD2 is the specific target of compound **20** in the LPS-induced inflammatory pathway.

**Figure 3:**
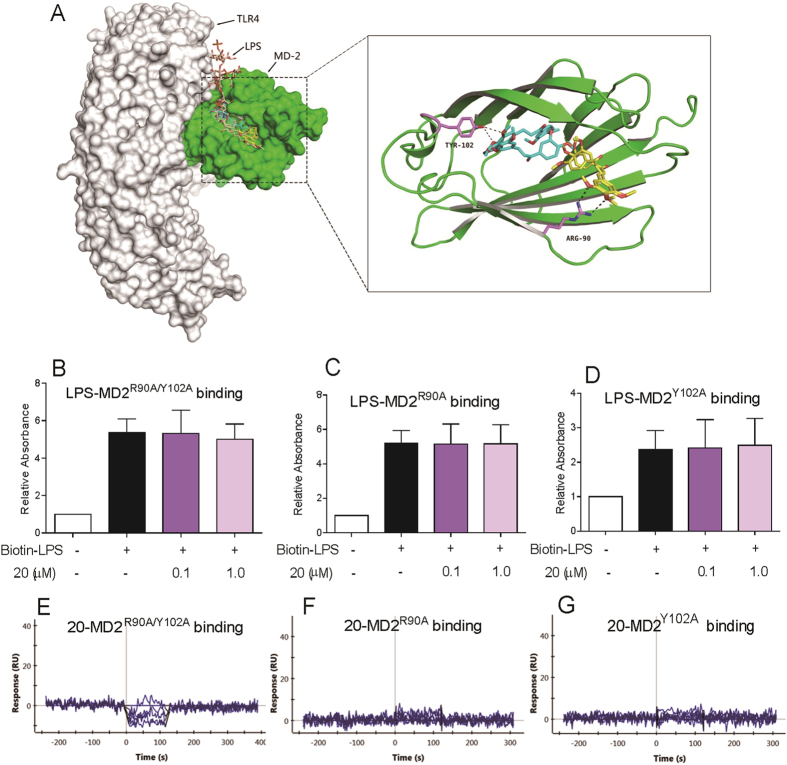
Compound **20** binds with MD2 via interacting with Arg^90^ and Tyr^102^. (**A**) Molecular docking of compound **20** with MD2 protein was carried out with the Sybyl-2.0 molecular modeling software from Tripos. Compound **20** did not inhibit biotin-LPS binding to (**B**) rhMD2^R90A/Y102A^, (**C**) rhMD2^R90A^ and (**D**) rhMD2^Y102A^ determined by ELISA. SPR analysis showed no interaction between compound **20** and (**E**) rhMD2^R90A/Y102A^, (**F**) rhMD2^R90A^ and (**G**) rhMD2^Y102A^ respectively. Data are mean values (±SEM) of at least three separate experiments.

In search of the potential mechanism of compound **20** binding to MD2 protein, we docked **20** to the crystal structure of the TLR4/MD2/LPS complex (PDB code: 3FXI) using molecular docking software. As shown in Fig. 3A, compound **20** fits into the hydrophobic pocket of MD2, overlapping a portion of the LPS binding sites. Moreover, **20** can form hydrogen bonds with the Arg^90^ and Tyr^102^ residues of the MD2 protein. To better understand the role of Arg^90^ and Tyr^102^ in **20** binding to MD2, we constructed three MD2 point mutation proteins (R90A mutation, Y102A mutation, and R90A and Y102A dual mutation) and examined the results. As shown in Fig. 3B–D, **20** had no effect on biotin-LPS binding to any of the three mutated MD2 proteins. SPR assay (Fig. 3E–G) also showed that **20** did not bind to MD2^R90A/Y102A^, MD2^R90A^, or MD2^Y102A^ mutations. These results elucidated the possible mechanism of compound **20** binding to MD2 via Arg^90^ and Tyr^102^ residues.

### Compound 20 inhibited LPS-induced activation of NF-κB and MAPKs in MPMs

**Figure 4:**
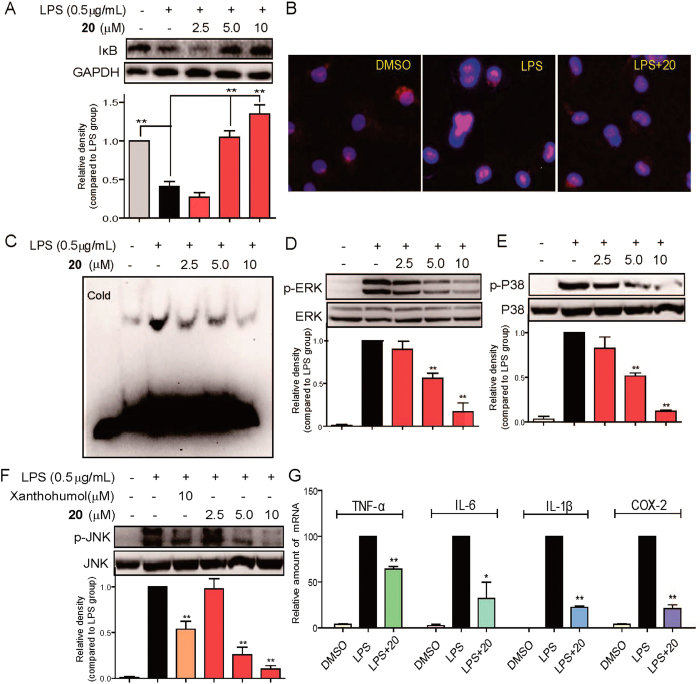
Effects of compound **20** on the LPS-induced activation of MAPK and NF-κB and expression of inflammatory genes. (**A**,**D,E**,**F**) MPMs pretreated with vehicle or compound **20** (2.5, 5, or 10 μM) or Xanthohumol (10 μM) for 30 min followed by incubation with LPS (0.5 μg/mL) for 20 min. The protein levels of IκB, GAPDH, p-ERK, ERK, p-P38, P38, p-JNK and JNK were measured by immunoblot analysis. (**B**) MPMs pretreated with vehicle or compound **20** (10 μM) for 30 min followed by incubation with LPS (0.5 μg/mL) for 1 h. Cells were subjected to fluorescence microscopy (200×), displaying the NF-κB P65-stained with Cy3-labeled antibody in pink and the nuclei-stained with 4,6-diamidino-2-phenylindole in blue. (**C**) MPMs pretreated with vehicle or compound **20** (2.5, 5, or 10 μM) for 30 min followed by incubation with LPS (0.5 μg/mL) for 1 h. Nuclear extracts were analyzed for NF-κB activity by EMSA. (**G**) MPMs pretreated with vehicle or compound **20** (10 μM) for 30 min followed by incubation with LPS (0.5 μg/mL) for 6 h. Expressions of TNF-α, IL-6, IL-1β, and COX-2 were analyzed with quantitative PCR (qPCR) using specific primers and normalization against the housekeeping gene β-actin. Data are mean values (±SEM) of 3–5 separate experiments. *p < 0.05 and **p < 0.01 vs. only-LPS stimulated group. The gels were run under the same experimental conditions. Shown are cropped gels/blots (The gels/blots of 4A and 4D-F with indicated cropping lines are shown in Supplementary Figure S8).

We evaluated the effects of compound **20** on the activation of downstream pro-inflammatory TLR4/MD2 signals, including NF-κB and MAPKs. As shown in Fig. 4A, compound **20** dose-dependently reversed LPS-induced IκBα degradation. As shown in Fig. 4B, LPS strengthened NF-κB p65 nuclear translocation (see the red point in the blue nucleus) while in **20**-pretreated MPMs, LPS-induced NF-κB p65 nuclear translocation was effectively inhibited. Electrophoretic mobility shift assay (EMSA) was also used to determine NF-κB transcriptional activation. Figure 4C shows that compound **20** dose-dependently inhibited DNA-binding activity of NF-κB. These findings confirmed the inhibitory ability of compound **20** on LPS-induced NF-κB activation. Regarding MAPK signaling, Fig. 4D–F shows that LPS stimulation for 20 min induced a significant phosphorylation of ERK, JNK, and P38, respectively, while pretreatment with compound **20** dose-dependently blocked LPS-induced MAPK phosphorylation in the MPMs. We also observed the effect of compound **20** on LPS-induced inflammatory gene transcription. As shown in Fig. 4G, compound **20** significantly inhibited LPS-induced up-regulation of TNF-α, IL-6, IL-1β, and COX-2 transcripts with statistical significance in the MPMs. These results altogether revealed that MD2 inhibitor **20** exhibited preventive influence in the TLR4/MD2 downstream pro-inflammatory signaling pathway.

### Compound 20 protects acute lung injury in rats by targeting MD2

**Figure 5:**
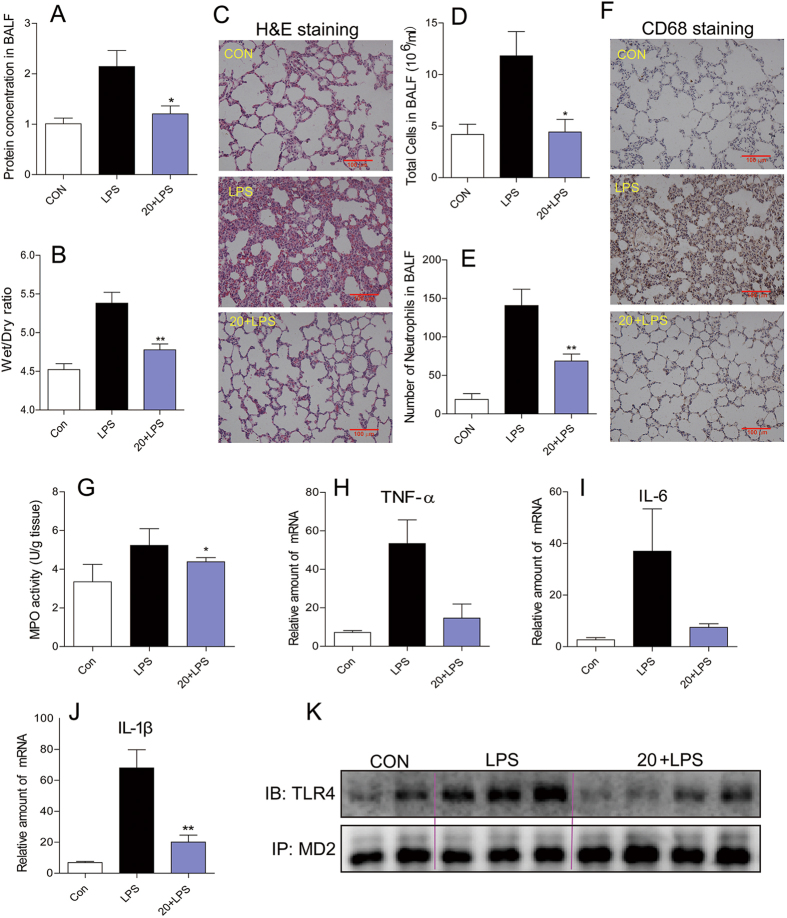
Effects of compound 20 on LPS-induced acute lung injury. SD rats were treated intragastrically with compound **20** at a dosage of 20 mg/kg b.wt for one week, then challenged with 5 mg/kg LPS. Rats were euthanized with ketamine after 6 h of LPS induction. (**A**) Protein concentration in BALF. (**B**) Lung Wet/Dry ratio. (**C**) Histopathological changes in lung tissues determined by H&E staining. (**D**,**E**) Number of total cells and neutrophils in BALF. (**F**) Macrophage infiltration in lung tissue measured by CD68 immunohistochemical staining. (**G**) Neutrophils activity in lung tissue determined by MPO activity. (**H**–**J**) Expression of inflammatory genes in lung tissue using QPCR. (**K**) Immunoprecipitation assay shows that compound **20** significantly reduced the formation of TLR4-MD2 complex in lung tissue induced by LPS. Data are mean values (±SEM) of 3–5 separate experiments. *p < 0.05 and **p < 0.01 vs. only-LPS stimulated group. The gels were run under the same experimental conditions. Shown are cropped gels/blots (Cropped gels/blots of 5 K with indicated cropping lines are also shown in Supplementary Figure S8).

In order to determine whether **20** was able to protect the test rats from ALI by targeting MD2 in response to intracheal LPS instillation, we first assessed the total protein concentrations in BALF, as well as the index of pulmonary edema by examining the lung wet/dry weight ratio. The BALF total protein concentrations were significantly increased after LPS administration compared to the control group (Fig. 5A). Administration of **20** evidently reduced the LPS-induced increase in protein concentrations in BALF. As illustrated in Fig. 5B, the lung wet/dry weight ratio was markedly higher in the LPS-treated group than the control group, and **20** treatment reduced LPS-induced pulmonary edema. LPS also caused observable lung histopathologic changes, including areas of inflammatory infiltration, hemorrhage, interstitial edema, thickening of the alveolar wall, and lung tissue destruction. These histopathological changes were ameliorated in the **20** treatment group (Fig. 5C).

Next, we evaluated the effect of **20** on LPS-induced inflammation in the lung tissue. LPS exposure caused an increase influx of total cells and neutrophils into BALF, and macrophage infiltration in the lung tissue (Fig. 5D–F), whereas **20** administration reduced this LPS-mediated neutrophil infiltration. Increased MPO activity reflects neutrophil infiltration in lung tissue; as shown in Fig. 5G, **20** decreased LPS-induced MPO activity in the lung tissue. LPS also increases inflammatory cytokine mRNA levels, and treatment with **20** significantly inhibited the LPS-induced mRNA expression of inflammatory cytokines in the lung tissue (Fig. 5H–J). Similar results were observed in the cultured human lung epithelial cell line Beas-2B (Supplementary Fig. S7).

We also confirmed the *in vivo* protective effect of compound **20** as associated with its MD2 inhibition. The level of TLR4/MD2 complex in lung tissues was first determined by immunoprecipitation assay. As shown in Fig. 5K, compared to the control group and **20**-treated group, LPS treatment significantly induced the formation of TLR4/MD2 complex in the rat lung tissues. This data confirming that **20** exhibited ALI-protective effect by targeting MD2 *in vivo*.

## Discussion

The main findings of this study include that we have developed novel chalcones with potent anti-inflammatory activities both *in vitro* and *in vivo*, and more importantly, that we demonstrated the biological mechanism and target for the anti-inflammatory action of compound **20**. Current research efforts on exploring MD2-targeting molecules to treat inflammatory diseases involve endogenous ligands, lipid-like antagonists, natural and synthetic chemicals^17^,^31^,^32^. In 2013, eritoran, the most attractive potential lipid-like agent for treating sepsis by targeting MD2, failed its phase III clinical study^33^. In recent years, more and more natural and synthetic chemicals as MD2 small molecular inhibitors were reported, including two new compounds, L6H21 and L48H37, which we investigated in our laboratory^20^,^21^,^22^,^23^,^34^,^35^. Although they showed positive results in treating inflammation and sepsis, none have entered clinical study. In addition, relatively little attention has been given to the structure-based drug design and medicinal chemistry of MD2 inhibitors, and the structure-activity relationship (SAR) of small-molecule MD2 inhibition remains unclear.

Here, the majority of synthesized chalcone derivatives showed inhibitory activity against LPS-induced TNF-α and IL-6 production in macrophages (Supplementary Fig. S2). Data from Supplementary Fig. S2 shows that compounds with the 3,4-OH group on A ring exhibited more potent anti-inflammatory activity against IL-6 than 3,4-OCH_3_ when there is the same substituent group in A ring. Meanwhile, when 3′,4′-OH group positioned in B ring, the influencing order of substituent group in A ring inhibited IL-6 production is 4-OCH_2_CH_3_ > 3,4-OCH_3_ > 3,4,5-OCH_3_ > 4-OCH_3_ > 3,4-F ≥ 2-F ≥ 2-Cl > 3-OH > 4-F > 3,5-F. For the inhibition of TNF-α, the activity will be increased when the 3,4-OCH_3_ group in A ring (except for compound **12**). Most importantly, compounds with electron withdrawing group 3,4-Cl in A ring show similar activity with compounds contain 3,4-OCH_3_ group in A ring (compound **22** *vs.* **14**). However, compound **20** contain 3,4,5-OCH_3_ group in A ring show increased inhibitory activity than compound **22**. This result indicates that the electron donating group in A ring is critical for their anti-inflammatory activity. The most active compound, **20**, was identified as a new MD2 inhibitor at both molecular and cellular levels. It directly binds with rhMD2 protein and competitively inhibits the interactions of both LPS-MD2 and MD2-TLR4. Given the MD2 inhibition, **20** blocked the activation of the TLR4/MD2 -downstream pro-inflammatory signaling pathway and, ultimately, cytokine overexpression induced by LPS. Although previously researched molecules such as JSH, xanthohumol, caffeic acid phenethyl ester, and curcumin have been confirmed to bind to MD2 protein, their specificity has not been demonstrated^20^,^21^,^22^,^23^. It is very important to note that we did not observe compound **20** to affect TLR4 protein, downstream kinase proteins, or MD2-independent TLR2 signaling, indicating a strong specificity for **20** targeting MD2.

We further investigated the underlying structural mechanism of **20** binding to the MD2 protein using molecular simulation. Although several non-lipid compounds have been reported to bind with the MD2 protein and their binding sites have been proposed, the X-ray diffraction-based structural information and exact binding mechanism are still unknown. The MD2 hydrophobic pocket is narrow and deep with a total surface area of 1000 Å^2^.^16^ The generous internal surface of the pocket is completely lined with hydrophobic residues, and the opening region of the pocket contains positively charged residues that facilitate the LPS binding^12^. The sites where natural and synthetic chemicals bind to the MD2 pocket were predicted by computer-assisted docking in this study. In previous studies, the residues Cys^133^, Lys^130^, Tyr^102^, Arg^90^, Ser^120^, Lys^122^, Phe^126^, and Gly^123^ in the MD2 pocket were considered to play a possible role in the interaction between the MD2 protein and natural small molecules such as xanthohumol, curcumin, JSH, and L6H21^20^,^21^,^22^,^34^,^36^. We found that the binding pattern of compound **20** with MD2 was the formation of hydrogen bonds at Arg^90^ and Tyr^102^ residues. This result somewhat confirms a prediction made by Peluso *et al.*, according to which isoxanthohumol is positioned at the deep hydrophobic interior of the MD2 pocket after making a hydrogen bond with Tyr^102^ residue.^20^ The present study’s data are also consistent with our previous report on another MD2-inhibitory chalcone L6H21.^34^ To validate our prediction, three mutations, MD2^R90A^, MD2^Y102A^, and MD2^R90A/Y102A^ proteins, were tested and the results clearly showed the importance of Arg^90^ and Tyr^102^ in MD2-**20** interaction (Fig. 3B–G). Thus, we not only identified a specific MD2 inhibitor in this study, but also clearly elucidated the binding sites.

Via targeting MD2, compound **20** inhibited LPS-induced MAPKs and NF-κB activation as well as inflammatory cytokine production in macrophages. These effects indicate the compound’s potential to protect lung tissue from LPS-induced inflammatory injuries *in vivo*. ALI is characterized by overwhelming inflammatory distress leading to pulmonary edema and infiltration^1^,^37^. Numerous pharmacological therapies for established ALI, including corticosteroids and steroids, have failed to prove beneficial in multicenter clinical trials^38^. Hadina *et al.* revealed that MD2-null mice display no response to pulmonary inflammation after nasal aspiration of LPS from *Neisseria meningitides*^39^. To this effect, MD2 is a potential therapeutic target to treat ALI. Although a number of chalcone derivatives have been identified as anti-inflammatory agents, few studies have demonstrated the efficiency of chalcones in treating ALI. In the present study, compound **20** was shown to attenuate LPS-induced lung injuries, accompanied by decreased pulmonary inflammation and TLR4/MD2 complex formation in lung tissue (Fig. 5). Non-lipid MD2 inhibitors and chalcone-derived anti-inflammatory agents could be considered, as such, effective agents for the treatment of ALI.

**Figure 6:**
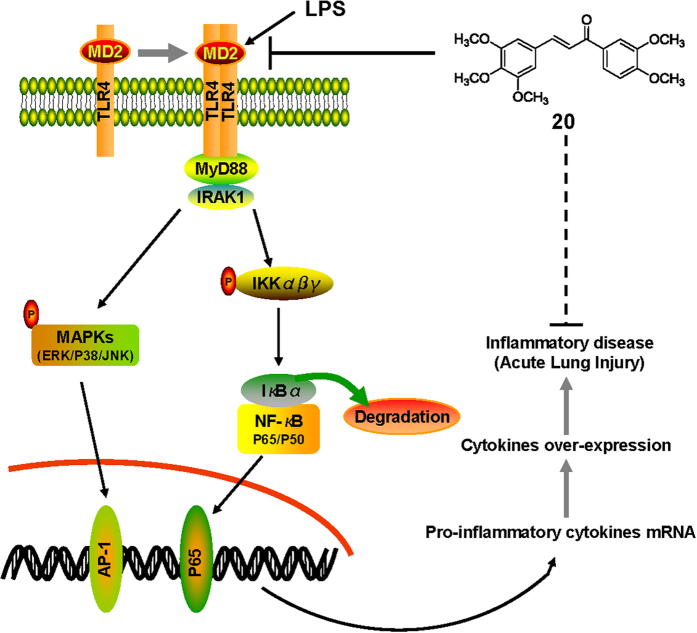
Proposed model of signaling pathway involved in compound 20 preventing LPS-induced TLR4 signaling pathway activation and acute lung injury.

In summary, among 31 synthesized chalcone derivatives, compound **20** exhibited the strongest anti-inflammatory activity. We tested compound **20** as a specific MD2 inhibitor and elucidated the binding mechanism of compound **20** binding to MD2. Our tests showed that compound **20** suppresses MAPK phosphorylation, NF-κB activation, and cytokine expression in LPS-stimulated macrophages; **20** exhibited protective effect on rat lung tissue after acute lung injury. A schematic for the protection of **20** from LPS-induced ALI is illustrated in Fig. 6. The new MD2 inhibitor, **20**, discovered in this study shows noteworthy potential as an ALI treatment agent. More importantly, our findings represent a new direction for the development of anti-inflammatory agents targeting MD2 from natural chalcone structures.

## Methods

### Chemical Synthesis

Solvents were distilled under positive pressure of dry argon before use and dried using standard methods. Triethylamine (Et_3_N) was distilled over calcium hydride and stored over potassium hydroxide. Unless otherwise noted, chemicals were obtained from local suppliers and were used without further purification. All reactions were monitored by thin-layer chromatography (250 μ silica gel 60 F_254_ glass plates). Visualization was performed by ultraviolet light methods and/or by staining with 3% ethanol solution of phosphomolybdic acid. Melting points were determined on a Fisher-Johns melting apparatus and were uncorrected. ^1^H-NMR and ^13^C-HMR spectra were recorded on a 600 or 500 MHz spectrometer (Bruker Corporation, Switzerland). The chemical shifts were recorded in parts per million with TMS as the internal reference. Electropray ionization mass spectra in positive mode (ESI-MS) data were measured on a Bruker Esquire HCT spectrometer. Column chromatography purifications were conducted out on silica gel 60 (E. Merck, 70−230 mesh). Thirty-one chalcone derivatives were synthesized by Claisen−Schmidt condensation between different substituted acetophenones and arylaldehydes. Chalcone derivatives with hydroxyl groups were synthesized by reflux in acidic medium using HCl as a catalyst, while other chalcones were synthesized at 5–8 °C in NaOH. All the reactions were monitored with silica gel thin layer chromatography. At the end of the reactions, water was added into the mixture to precipitate the products. All compounds were purified with silica gel column chromatography. The yields of products were 10−95% after purification. Their structures were characterized by spectral data from HRMS, ESI-MS, ^1^H-NMR and ^13^C-NMR. The spectral data of novel or unreported compounds are exhibited in Supplementary Information.

### Reagents

Chemical reagents, Pam3CK and lipopolysaccharide (LPS) were purchased from Sigma (Sigma, St. Louis, MO, USA). RPMI-1640, DMEM medium and FBS were obtained from Gibco (Gibco, Eggenstein, Germany). EBioscience (eBioScience, San Diego, CA, USA) was the source of mouse IL-6 ELISA Kit, mouse TNF-α ELISA Kit and anti-rhMD2 antibody. Trizol-reagent was purchased from Invitrogen (Invitrogen, Carlsbad, CA, USA). Two-step M-MLV Platinum SYBR Green qPCR SuperMix-UDG kit (Invitrogen, Carlsbad, CA). Chemiluminescent EMSA Kit, ECL detection reagent, protein A + G agarose, biotin-labeled NF-κB probe and Cellular NF-κB p65 Translocation Kit were obtained from Beyotime Biotechnology (Beyotime Biotech, Nantong, China). Anti-GAPDH, anti-IκBα, anti-p-ERK, anti-ERK, anti-CD68, anti-TLR4 and anti-MD2 antibody were purchased from Santa Cruz Biotechnology (Santa Cruz, CA, USA); anti-p-P38, anti-P38, anti-p-JNK and anti-JNK were from Cell Signaling Technology (Cell Signaling Technology, Danvers, MA, USA). Recombinant human MD2 (rhMD2) protein, Recombinant human TLR4 (rhTLR4) protein, and TLR2 inhibitor CU-CPT22 were purchased from R&D Systems, Inc. (Minneapolis, MN, USA). Mutated rhMD2 was obtained using the methods described in our previous publication.^35^

### Animals

Male ICR mice weighing 18–22 g and male SD rats weighing 180–200 g were obtained from the Wenzhou Medical University Animal Center. Animals were housed at constant room temperature with a 12:12 hour light-dark cycle, and fed with a standard rodent diet and water. The animals were acclimatized to the laboratory for at least 7 days before use in experiments. Protocols involving the use of the animals were approved by the Wenzhou Medical University Animal Policy and Welfare Committee (Approval documents: wydw2014-0001). All animal care and experiments were performed in accordance with the approved protocols and the ‘The Detailed Rules and Regulations of Medical Animal Experiments Administration and Implementation’ (Document No. 1998-55, Ministry of Public Health, China).

### Cells

Mouse RAW 264.7 macrophages were obtained from the American Type Culture Collection (ATCC, USA). Cells were grown in DMEM medium supplemented with 10% FBS, 100 U/mL penicillin, and 100 mg/mL streptomycin at 37 °C with 5% CO_2_. Mouse primary peritoneal macrophage (MPM) preparation was conducted as described above^40^. Briefly, ICR mice were stimulated by intraperitoneal (ip) injection of 1.5 mL of thioglycollate solution per mouse and kept in pathogen-free conditions for 3 days before peritoneal macrophage isolation. Total peritoneal macrophages were harvested by washing the peritoneal cavity with 8 mL RPMI-1640 medium per mouse, centrifuging, then re-suspending the pellet in RPMI-1640 medium with 10% FBS, 100 U/mL penicillin, and 100 mg/mL streptomycin. Non-adherent cells were removed by washing with medium at 4 h after seeding. Experiments were conducted once the cells adhered firmly to the culture plates.

### Detection of TNF-α and IL-6

After cell treatment, cultural medium were collected and the levels of inflammatory cytokines (TNF-α and IL-6) in the medium were determined using an ELISA kit as previously described^41^. The total amount of the inflammatory factor in the medium was normalized to the total protein quantity of the viable cell pellets.

### Real-time Quantitative PCR

Total RNA was isolated from cells using Trizol-reagent and quantified by UV absorption at 260 and 280 nm. Both reverse transcription and quantitative PCR were performed using a two-step M-MLV Platinum SYBR Green qPCR SuperMix-UDG kit. An Eppendorf Mastercycler ep realplex detection system (Eppendorf, Hamburg, Germany) was used for q-PCR analysis. The primers of genes, including TNF-α, IL-6, IL-1β, COX-2, and β-actin, were synthesized by Invitrogen. The amount of each gene was determined and normalized by the amount of β-actin.

### Cellular NF-κB p65 Translocation Assay

MPMs were treated with LPS (0.5 μg/mL) in the presence or absence of compound **20** for 1 h. The cells were immunofluorescence-labeled according to the manufacturer’s instructions using a Cellular NF-κB p65 Translocation Kit as described previously^42^. P65 protein and nuclei, which fluoresce as red and blue, respectively, were simultaneously viewed under a fluorescence microscope (200X; Nikon, Tokyo, Japan) at an excitation wavelength of 350 nm for DAPI and 540 nm for cyanine 3 (Cy3). The red and blue images were overlaid to create the dual-color images shown below.

### Western Blot Assay

The Western Blot assay was conducted as previously described^43^. Briefly, MPM cell lysates prepared in standard cell lysis buffer were gel separated and proteins were transferred to PVDF membranes (Bio-rad Laboratories, Richmond, CA, USA). Membranes were then probed with primary antibodies and secondary HRP-conjugated antibodies and developed using ECL detection reagent (Beyotime Biotech, Nantong, China).

### Electrophoretic Mobility Shift Assay (EMSA)

EMSA was performed with a non-radioactive (biotin label) gel shift assay according to the manufacturer’s instructions as previously described^34^. Briefly, 5 μg nuclear extract was incubated with 2 nM biotin-labeled NF-κB probe in binding buffer for 30 min at room temperature. The reaction mixtures were then separated in a 4% nondenaturing polyacrylamide gel in 0.5 × TBE at 100 V for 1 h. The DNA/protein complex was then transferred to a nylon membrane, conjugated with Streptavidin-HRP, and visualized using ECL detection reagent.

### Fluorescence Spectroscopy Assay

Fluorescence measurements were performed with a spectraMax M5 (Molecular Devices, Sunnyvale, CA) as previously described^34^. Briefly, rhMD2 protein (5 nM) and 1,1′-Bis(anilino)- 4,4′-bis (naphthalene) −8,8′-disulfonate (bis-ANS, 5 μM) were mixed in PBS (pH 7.4) and incubated until reaching stable relative fluorescence units (RFUs) emitted at 430–590 nm under excitation at 385 nm at 25 °C in a 1 cm path-length quartz cuvette. Nonfluorescent **20** (at 5, 10, 20, or 30 μM) was then treated for 5 min, followed by relative fluorescence unit (RFU) measurement at 420–600 nm.

### Surface Plasmon Resonance Analysis (SPR)

The binding affinities of **20** with rhMD2, rhTLR4, MD2^R90A/Y102A^, MD2^R90A^, and MD2^Y102A^ proteins were determined using a ProteOn XPR36 Protein Interaction Array system (Bio-Rad Laboratories, Hercules, CA, USA) with an HTE sensor chip (ProteOn™, #176-5033). Briefly, protein was loaded to the sensors activated with 10 mM NiSO_4_. The compound **20** samples (at 6.25, 12.5, 25, 50, or 100 μM) were prepared with running buffer (PBS, 0.1% SDS, 5% DMSO). Sensor and sample plates were placed on the instrument, then the compound **20** samples were captured in flow cell one, leaving the second flow cell as a blank. Five concentrations were injected simultaneously at a flow rate of 30 μM/min for a 120 s association phase, followed by a 120 s dissociation phase at 25 °C. The final graphs were obtained by subtracting blank sensorgrams from the duplex or quadruplex sensorgrams. Data were analyzed with ProteOn manager software. KD was calculated by global fitting of the kinetic data from various concentrations of **20** using a 1:1 Langmuir binding model.

### MD2 Competitive ELISA

The competitive effects of compound **20** on LPS binding to rhMD2, MD2^R90A/Y102A^, MD2^R90A^, and MD2^Y102A^ protein were determined by ELISA. Briefly, rhMD2 antibody was coated to a 96-well plate at 4 °C overnight, then rhMD2 (4 μg/mL) in 10 mM Tris-HCl buffer was added to the pre-coated plate for 1.5 h at room temperature. After washing with PBST, biotin-labeled LPS was added to the plate with or without the presence of compound **20** (0.1 and 1.0 μM), resulting in absorbance values at 450 nm (A450). Data were shown as related values compared to the vehicle control group (Mean±SEM, n=4 separate determinations).

### Docking of 20 to TLR4/MD2/LPS Complex

Docking simulation of **20** withTLR4-MD2-LPS complex (PDB code 3FXI) was performed using molecular modeling packages in Sybyl-2.0 (Tripos, St. Louis, MO, USA). Energy minimization of modeled structures was performed using the Tripos force field with Gasteiger-Hückel charges, a fixed dielectric constant of 80, and a non-bonded cutoff radius of 8 Å. Minimization was carried out for a maximum of 5,000 iterations subject to a termination gradient of 0.05 kcal/(mol·Å). The ligand-binding groove on the TLR4/MD2/LPS complex was kept rigid, whereas all torsible bonds of **20** were set free to allow flexible docking to produce more than 100 structures. Final docked conformations were clustered within the tolerance of 1 Å root-mean-square deviation.

### Immunoprecipitation

MPMs were treated with LPS (0.5 μg/mL) for 5 min in the presence or absence of 10 μM compound **20**. Cells were lysed with an extraction buffer (containing mammalian protein extraction reagent supplemented with protease and phosphatase inhibitor cocktails) and centrifuged at 12,000 rpm for 10 min at 4 °C. A sufficient amount of MD2 antibody was added to 400 μg protein and gently rotated at 4 °C overnight. The immunocomplexes were collected with protein A + G agarose, and the precipitates were washed four times with ice-cold PBS. The proteins were then released by boiling in sample buffer, followed by Western blot analysis as described above.

### Flow Cytometry

Mouse RAW264.7 macrophages were starved for 3 h before experimentation. Cells were incubated with or without FITC-LPS (50 μg/mL) in the presence or absence of **20** (0.1, 1 and 10 μM) for 30 min. After incubation, macrophages were fixed with paraformaldehyde for 10 min at 4 °C and washed with PBS before being analyzed by flow cytometry.

### LPS-induced ALI

Male Sprague Dawley (SD) rats were randomly divided into three groups, designated “control” (5 rats, only received the vehicle of 0.9% saline), “LPS” (7 rats, received 5 mg/kg LPS alone) and “**20** + LPS” (6 rats, received both compound **20** and 5 mg/kg LPS). Prior to LPS-induced ALI, the 20 + LPS group rats were treated intragastrically with **20** at a dosage of 20 mg/kg/day continuously for one week. Under ether anesthesia, all the rats were exposed their trachea and challenged with intratracheal instillation of 50 μL of LPS (*E. coli* 055:B5; 5 mg/kg, dissolved in 0.9% saline), while the control group challenged with intratracheal instillation of 50 μL of 0.9% saline. Rats were then euthanized with ketamine after 6 h of LPS induction. The chest cavity of each animal was carefully opened and bronchoalveolar lavage fluid (BALF) was collected.

### BALF Analysis

The collected BALF was centrifuged at 1000 rpm for 10 min at 4 °C, then the supernatant was used for protein concentration detection and subsequent cytokine determination. The precipitation was re-suspended using 50 μL physiological saline. The total number of cells on BALF were detected with a cell counting instrument. The number of neutrophils on BALF were examined using Wright-Gimesa stain.

### Lung Wet/Dry Ratio

To observe the pulmonary edema, the lung wet/dry weight ratio was calculated. After the middle lobe of right lung was collected, the wet weight was recorded. Lung were then heated in a thermostatic oven at 65 °C for 72 h and weighed to determine the baseline lung dry mass levels.

### Histopathological Study

The superior lobe of the right lung was collected and fixed in 4% paraformaldehyde, then embedded in paraffin and cut into 5 μm sections. The sections were stained with hematoxylin and eosin using standard protocol for light microscopy examination.

### Immunohistochemistry

Tissue sections (5 μm thickness) were prepared, deparaffinized in xylene, and hydrated using an ethanol gradient. A Pressure-cooker was used for heat- induced antigen retrieval (10 mM sodium citrate buffer, pH 6.5). After treatment with 30% of hydrogen peroxide, all sections were blocked in 5% bovine serum albumin (BSA) and incubated with primary anti-CD68 antibody overnight at 4 °C. The slides were then incubated with HRP-labeled secondary antibody for 10 min. After the sections were incubated with 3,3-diaminobenzidine tetrahydrochloride (DAB) for color development and counterstained with hematoxylin, the slides were evaluated under a microscope.

### Statistical analysis

Data collected from experiments were analyzed using Graphpad prism 6.0 software. Values were expressed as mean ± SEM. One way ANOVA test was employed to analyze the differences between sets of data. A p-value of <0.05 was considered as statistically significant and denoted as*. *In vitro* experiments were performed with n ≥ 3 independent repeats. *In vivo* experiments were performed with n ≥ 5 rats in each group.

## Additional Information

**How to cite this article**: Zhang, Y. *et al.* Discovery of new MD2 inhibitor from chalcone derivatives with anti-inflammatory effects in LPS-induced acute lung injury. *Sci. Rep.* **6**, 25130; doi: 10.1038/srep25130 (2016).

## Supplementary Material

Supplementary Information
