# Supplementary material for: Discovery of new MD2 inhibitor from chalcone derivatives with anti-inflammatory effects in LPS-induced acute lung injury
Source: Sci Rep. 2016 Apr 27;6:25130. doi: 10.1038/srep25130 (PMC4846832; doi:10.1038/srep25130)
Supplement: Supplementary Information [file srep25130-s1.doc]

*Supplementary Information*

**Discovery of new MD2 inhibitor from chalcone derivatives with anti-inflammatory effects in LPS-induced acute lung injury**

Yali Zhang^1^, Jianzhang Wu^1^, Shilong Ying^1^, Gaozhi Chen^1^, Beibei Wu^2^, Tingting Xu^2^, Zhiguo Liu^1^, Xing Liu^1^, Lehao Huang^1^, Xiaoou Shan^2^, Yuanrong Dai^2^, Guang Liang^1,*^

1. Chemical Biology Research Center at School of Pharmaceutical Sciences, Wenzhou Medical University, Wenzhou, Zhejiang 325035, China

2. The 2^nd^ Affiliated Hospital, Wenzhou Medical University, Wenzhou, Zhejiang 325035, China

* Corresponding author:

Guang Liang, Ph.D, Professor

Director, Chemical Biology Research Center

School of Pharmaceutical Sciences, Wenzhou Medical University

Tel: (+86)-577-86699396; Fax: (+86)-577-86699396

E-mail: wzmcliangguang@163.com

**1. Chemical structures of current reported MD2 inhibitors**

**
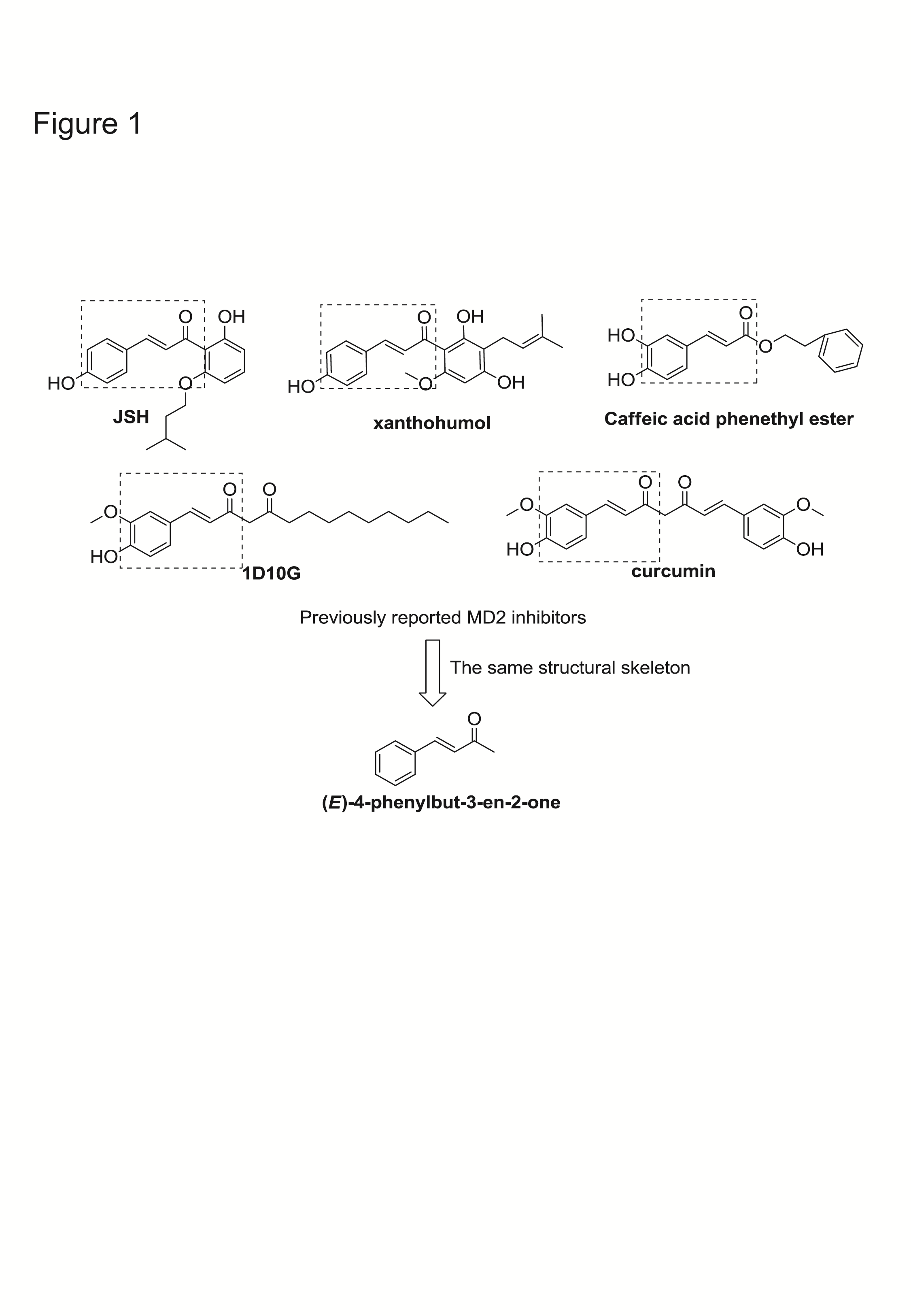
**

**Supplementary Figure S1.** Chemical structures of current reported MD2 inhibitors.

**2. The spectral data of synthesized compounds**

HPLC condition for purity determination: column: Agilent Eclipse XDB C18 5 μm 4.6 mm × 150 mm, flow: 1 mL/min, UV: 320 nm, water/acetonitrile: 0-5 min: 80/20, 5-6 min: 80/20 to 20/80, 6-15 min, 20/80.

The original ^1^H NMR spectra of compounds 1-31 were shown in Supplementary Figure S3.

*(E)*-3-(3,4-Dihydroxyphenyl)-1-(4-methoxyphenyl)prop-2-en-1-one (**1**)

Red-brown power, 45.5% yield, mp 170.4~172.9 °C [172-178 °C Lit^1^], HPLC purity 95.4% (r.t. 8.233 min). ^1^H-NMR (500MHz, DMSO) δ: 8.12 (d, *J* = 8.5 Hz, 2H), 7.63 (d, *J* = 15.5 Hz, 1H), 7.56 (d, *J* = 15.5 Hz, 1H), 7.26 (d, *J* = 1.5 Hz, 1H), 7.18 (dd, *J_1_* = 1.5 Hz, *J_2_* = 8.5 Hz, 1H), 7.07 (d, *J* = 8.5 Hz, 2H), 6.81 (d, *J* = 9.0 Hz, 1H), 3.86 (s, 3H). ESI-MS m/z: 270.9 (M+H)^+^. HRMS (ESI): calcd for C_16_H_14_O_4_ (MH+) 271.0965; found 271.0965.

*(E)*-3-(3,4-Dimethoxyphenyl)-1-(4-methoxyphenyl)prop-2-en-1-one (**2**)

Yellow power, 18.27% yield, mp 80.0~82.3 °C [90-92 °C Lit^2^], HPLC purity 97.8% (r.t. 8.972 min). ^1^H-NMR (600 MHz, CDCl_3_) δ: 8.04 (d, *J* = 8.4 Hz, 2H), 7.76 (d, *J* = 15.6 Hz, 1H), 7.41 (d, *J* = 15.6 Hz, 1H), 7.24 (dd, *J_1_* = 1.2 Hz, *J_2_* = 8.4 Hz, 1H), 7.16 (d, *J* = 1.2 Hz, 1H), 6.99 (d, *J* = 9.0 Hz, 2H), 6.92 (d, *J* = 8.4, 1H), 3.96 (s, 3H), 3.94 (s, 3H), 3.89 (s, 3H). ESI-MS m/z: 299.2 (M+H)^+^. HRMS (ESI): calcd for C_18_H_18_O_4_ (MH+): 299.1278; found 299.1277.

*(E)*-3-(3,4-Dihydroxyphenyl)-1-(4-ethoxyphenyl)prop-2-en-1-one (**3**)

Yellow power, 66.51% yield, mp 168.7~170.3 °C. ^1^H-NMR (600 MHz, DMSO) δ: 8.12 (d, *J* = 7.2 Hz, 2H), 7.65 (d, *J* = 15.6 Hz, 1H), 7.62 (d, *J* = 15.6 Hz, 1H), 7.32 (s, 1H), 7.19 (d, *J* = 8.4 Hz, 1H), 7.04 (d, *J* = 7.2 Hz, 2H), 6.90 (d, *J_1_* = 1.8 Hz, *J_2_* = 8.4 Hz, 1H), 4.17 (q, *J* = 7.2 Hz, 2H), 1.41 (t, *J* = 7.2 Hz, 3H). ESI-MS m/z: 283.0 (M-H)^-^. HRMS (ESI): calcd for C_16_H_14_O_4_ (MH+): 285.1122; found 285.1109.

*(E)*-3-(3,4-Dimethoxyphenyl)-1-(4-ethoxyphenyl)prop-2-en-1-one (**4**)

Yellow power, 67.63% yield, mp 88.9~93.4 °C, HPLC purity 98.6% (r.t. 9.466 min). ^1^H-NMR (600 MHz, CDCl_3_) δ: 8.03 (d, *J* = 8.4 Hz, 2H), 7.75 (d, *J* = 15.6 Hz, 1H), 7.41 (d, *J* = 15.6 Hz, 1H), 7.23 (dd, *J_1_* = 1.8 Hz, *J_2_* = 8.4 Hz, 1H), 7.16 (d, *J* = 2.4 Hz, 1H), 6.97 (d, *J* = 9.0 Hz, 2H), 6.90 (d, *J* = 8.4 Hz, 1H), 4.12 (q, *J* =7.2 Hz, 2H), 3.96 (s, 3H), 3.93 (s, 3H), 4.58 (t, 5H). ESI-MS m/z: 313.2 (M+H)^+^. HRMS (ESI): calcd for C_19_H_20_O_4_ (MH+): 313.1435; found 313.1446.

*(E)*-1-(2-Chlorophenyl)-3-(3,4-dihydroxyphenyl)prop-2-en-1-one (**5**)

Brown power, 81.61% yield, mp 154.4~156.0 °C [174-178 °C Lit^1^]. ^1^H-NMR (600 MHz, DMSO-*d_6_*) δ: 8.32 (brs, 2H), 7.50~7.54 (m, 3H), 7.45~7.49 (m, 1H), 7.32 (d, *J* = 16.2 Hz, 1H), 7.23 (d, *J* = 2.4 Hz, 1H), 7.09 (dd, *J_1_* = 1.8 Hz, *J_2_* = 8.4 Hz, 1H), 6.96 (d, *J* = 16.2 Hz, 1H), 6.99 (d, *J* = 8.4 Hz, 1H). ESI-MS m/z: 275.2 (M+H)^+^. HRMS (ESI): calcd for C_15_H_11_ClO_3_ (MH+): 275.0470; found 275.0476.

*(E)*-1-(2-Chlorophenyl)-3-(3,4-dimethoxyphenyl)prop-2-en-1-one (**6**)

Light Yellow power, 92.38% yield, mp 94.5~96.0 °C, HPLC purity 96.2% (r.t. 9.193 min). ^1^H-NMR (600 MHz, CDCl_3_) δ: 7.45~7.47 (m, 2H), 7.42 (dt, *J_1_* = 1.8 Hz, *J_2_* = 7.8Hz, 1H), 7.38 (d, *J* = 16.2 Hz, 1H), 7.36 (dt, *J_1_* = 1.2 Hz, *J_2_* = 7.8 Hz, 1H), 7.14 (dd, *J_1_* = 1.8 Hz, *J_2_* = 8.4Hz, 1H), 7.09 (d, *J* = 2.4 Hz, 1H), 6.99 (d, *J* = 16.2 Hz, 1H), 6.88 (d, *J* = 8.4 Hz, 1H), 3.95 ( s, 6H). ESI-MS m/z: 303.3 (M+H)^+^. HRMS (ESI): calcd for C_17_H_15_ClO_3_ (MH+): 303.0783; found 303.0767.

*(E)*-3-(3,4-Dihydroxyphenyl)-1-(4-fluorophenyl)prop-2-en-1-one (**7**)

Yellow power, 78.84% yield, mp 224.4~226.0 °C, HPLC purity 98.5% (r.t. 8.175 min). ^1^H-NMR (600 MHz, Acetone-*d_6_*) δ: 8.35 (brs, 2H), 8.19~8.23 (m, 2H), 7.69 (d, *J* = 15.6 Hz, 1H), 7.62 (d, *J* = 15.6 Hz, 1H), 7.34 (d, *J* = 1.8 Hz, 1H), 7.28~7.32 (m, 2H), 7.21 (dd, *J_1_* = 1.8 Hz, *J_2_* = 7.8 Hz, 1H), 6.91 (d, *J* = 8.4 Hz, 1H). ESI-MS m/z: 258.9 (M+H)^+^. HRMS (ESI): calcd for C_15_H_11_FO_3_ (MH+): 259.0765; found 259.0765.

*(E)*-3-(3,4-Dimethoxyphenyl)-1-(4-fluorophenyl)prop-2-en-1-one (**8**)

Yellow powder, 91.03% yield, mp 76.7~79.4 °C. ^1^H-NMR (600 MHz, CDCl_3_) δ: 8.05 (dd, *J_1_* = 5.4 Hz, *J_2_* = 8.4 Hz, 2H), 7.77(d, *J* = 15.6 Hz, 1H), 7.36 (d, *J* = 15.6 Hz, 1H) 7.24 (dd, *J_1_* = 1.8 Hz, *J_2_* = 8.4 Hz, 1H), 7.16~7.19 (m, 2H), 7.16 (d, *J* = 2.4 Hz, 1H), 6.91 (d, *J* = 8.4 Hz, 1H), 3.96 (s, 3H), 3.94 (s, 3H). ESI-MS m/z: 287.0 (M+H)^+^. HRMS (ESI): calcd for C_17_H_15_FO_3_ (MH+): 287.1078; found 287.1078.

*(E)*-1-(3,4-Difluorophenyl)-3-(3,4-dihydroxyphenyl)prop-2-en-1-one (**9**)

Yellow power, 77.90% yield, mp 234.9 °C Decompose. ^1^H-NMR (600 MHz, DMSO-*d_6_*) δ: 9.80 (s, 1H), 9.12 (s, 1H), 8.18~8.21 (m, 1H), 8.02~8.04 (m, 1H), 7.60~7.67 (m, 1H), 7.65 (d, *J* = 16.2 Hz, 1H), 7.63 (d, *J* = 16.2 Hz, 1H), 7.30 (d, *J* = 2.4 Hz, 1H), 7.22 (dd, *J_1_* = 2.4 Hz, *J_2_* = 8.4 Hz, 1H), 6.82 (d, *J* = 8.4 Hz, 1H). ESI-MS m/z: 277.1 (M+H)^+^. HRMS (ESI): calcd for C_15_H_10_F_2_O_3_ (MH+): 277.0671; found 277.0681.

*(E)*-1-(3,4-Difluorophenyl)-3-(3,4-dimethoxyphenyl)prop-2-en-1-one (**10**)

Yellow power, 74.99% yield, mp 118.6~120.4 °C, HPLC purity 100.0% (r.t. 9.560 min). ^1^H-NMR (600 MHz, CDCl_3_) δ: 7.85~7.88 (m, 1H), 7.80~7.81 ( m, 1H), 7.79 (d, *J* = 15.6 Hz, 1H), 7.31 (d, *J* = 15.6 Hz, 1H), 7.26~7.31 (m, 1H), 7.25 (dd, *J_1_* = 1.8 Hz, *J_2_* = 8.4 Hz, 1H), 7.16 (d, *J* = 1.8 Hz, 1H), 6.91 (d, *J* = 7.8 Hz, 1H), 3.98 (s, 3H), 3.94 (s, 3H). ESI-MS m/z: 305.3 (M+H)^+^. HRMS (ESI): calcd for C_17_H_14_F_2_O_3_ (MH+): 305.0984; found 305.0984.

*(E)*-3-(3,4-Dihydroxyphenyl)-1-(3,4,5-trimethoxyphenyl)prop-2-en-1-one (**11**)

Kelly syrupy product, 98.76% yield, mp 129.9~132.1 °C [153-154 °C, Lit^3^]. ^1^H-NMR (600 MHz, DMSO-*d_6_*) δ: 9.13 (s, 2H), 7.64 (d, *J* = 15.6 Hz, 1H), 7.60 (d, *J* = 15.6 Hz, 1H), 7.39 (s, 2H), 7.30 (d, *J* = 1.8 Hz, 1H), 7.22 (dd, *J_1_* = 1.8 Hz, *J_2_* = 8.4 Hz, 1H), 6.82 (d, *J* = 8.4 Hz, 1H), 3.89 (s, 6H), 3.84 (s, 3H). ESI-MS m/z: 331.1 (M+H)^+^. HRMS (ESI): calcd for C_18_H_18_O_6_ (MH+): 331.1176; found 331.1176.

*(E)*-3-(3,4-Dimethoxyphenyl)-1-(3,4,5-trimethoxyphenyl)prop-2-en-1-one (**12**)

Light yellow power, 25.46% yield, mp 126.5~128.2 °C Lit^4^. ^1^H-NMR (600 MHz, CDCl_3_) δ: 9.86 (d, *J* = 15.6 Hz, 1H), 7.33 (d, *J* = 15.6 Hz, 1H), 7.26 (s, 2H), 7.260 (dd, *J_1_* = 1.8 Hz, *J_2_* = 8.4 Hz, 1H), 7.16 (d, *J* = 1.8 Hz, 1H), 6.92 (d, *J* = 8.4 Hz, 1H), 3.94 (s, 6H), 3.92 (s, 3H), 3.90 (s, 3H), 3.89 (s, 3H). ESI-MS m/z: 359.2 (M+H)^+^. HRMS (ESI): calcd for C_20_H_22_O_6_ (MH+): 359.1489; found 359.1789.

*(E)*-3-(3,4-Dihydroxyphenyl)-1-(3,4-dimethoxyphenyl)prop-2-en-1-one (**13**)

Yellow power, 72.59% yield, mp 93.8~95.4 °C [132-137 °C Lit^3^]. ^1^H-NMR (600 MHz, Acetone-*d_6_*) δ: 7.84 (dd, *J_1_* = 1.8 Hz, *J_2_* = 8.4 Hz, 1H), 7.67 (d , *J* = 15.6 Hz, 1H), 7.66 (d, *J* = 1.8 Hz, 1H), 7.63 (d, *J* = 15.6 Hz, 1H), 7.32 (d , *J* = 1.8 Hz, 1H), 7.17 (dd, *J_1_* = 1.8 Hz, *J_2_* = 8.4 Hz, 1H), 7.09 (d, *J* = 8.4 Hz, 1H), 7.90 (d, *J* = 8.4 Hz, 1H), 3.892 (s, 6H). ESI-MS m/z: 301.2 (M+H)^+^. HRMS (ESI): calcd for C_17_H_16_O_5_ (MH+): 301.1071; found 301.1082.

*(E)*-1,3-bis(3,4-Dimethoxyphenyl)prop-2-en-1-one (**14**)

Light yellow power, 57.52% yield, mp 105.5~107.2 °C [102-104 °C Lit^5^]. ^1^H-NMR (600 MHz, CDCl_3_) δ: 7.77 ( d, *J* = 15.6 Hz, 1H), 7.69 ( dd , *J_1_* = 1.8 Hz, *J_2_* = 8.4 Hz, 1H), 7.63 (d, *J* = 1.8 Hz, 1H), 7.42 (d, *J* = 15.6 Hz, 1H), 7.25 ( dd , *J_1_* = 1.8 Hz, *J_2_* = 8.4 Hz, 1H), 7.17 (d, *J* = 1.8 Hz, 1H), 6.94 (d, *J* = 8.4 Hz, 1H), 6.91 (d, *J* = 8.4 Hz, 1H), 3.98 ( s, 3H), 3.97 (s, 3H), 3.96 (s, 3H), 3.94 (s, 3H). ESI-MS m/z: 330.2 (M+H)^+^. HRMS (ESI): calcd for C_19_H_20_O_5_ (MH+): 329.1384; found 329.1403.

*(E)*-1-(3,5-Difluorophenyl)-3-(3,4-dihydroxyphenyl)prop-2-en-1-one (**15**)

Yellow powder, 46.57% yield, mp 240.2 °C decompose. ^1^H-NMR (600 MHz, Acetone-*d_6_*) δ: 7.73~7.76 (m, 3H), 7.67 (d, *J* = 15.6 Hz, 1H), 7.38 (d, *J* = 1.8 Hz, 1H), 7.30 (tt, *J_1_* = 2.4 Hz, *J_2_* = 8.4 Hz, 1H), 7.25 (dd, *J_1_* = 1.8 Hz, *J_2_* = 7.8 Hz, 1H), 6.91 (d, *J* = 7.8 Hz, 1H). ESI-MS m/z: 277.1 (M+H)^+^. HRMS (ESI): calcd for C_15_H_10_F_2_O_3_ (MH+): 277.0671; found 277.0671.

*(E)*-1-(3,5-Difluorophenyl)-3-(3,4-dimethoxyphenyl)prop-2-en-1-one (**16**)

Yellow power, 54.71% yield, mp 107.4~109.2 °C. ^1^H-NMR (600 MHz, CDCl_3_) δ: 7.80 (d, *J* = 15.6 Hz, 1H), 7.50~7.53 (m, 2H), 7.27 (d, *J* = 15.6 Hz, 1H), 7.25 (d, *J* = 7.8 Hz, 1H), 7.16 (d, *J* = 1.8 Hz, 1H), 7.03 (tt, *J_1_* = 1.2 Hz, *J_2_* = 8.4 Hz, 1H), 6.92 (d, *J* = 7.8 Hz, 1H), 3.97 (s, 3H), 3.95 (s, 3H). ESI-MS m/z: 305.0 (M+H)^+^. HRMS (ESI): calcd for C_17_H_14_F_2_O_3_ (MH+): 305.0984; found 305.0987.

*(E)*-3-(3,4-Dihydroxyphenyl)-1-(3-hydroxyphenyl)prop-2-en-1-one (**17**)

Yellow power, 57.45% yield, mp 193.6~195.4 °C [191-192 °C Lit^1^]. ^1^H-NMR (600 MHz, Acetone-*d_6_*) δ: 8.64 (brs, 1H), 8.53 (brs, 1H), 8.18 (brs, 1H), 7.66 (d, *J* = 15.6 Hz, 1H), 7.61 (d, *J* = 7.8 Hz, 1H), 7.55 (d, *J* = 15.6 Hz, 1H), 7.53 (s, 1H), 7.37 (t, *J* = 7.8 Hz, 1H), 7.33 (s, 1H), 7.20 (d, *J* = 7.8 Hz, 1H), 7.09 (d, *J* = 7.8 Hz, 1H), 6.91 (d, *J* = 7.8 Hz, 1H). ESI-MS m/z: 257.0 (M+H)^+^. HRMS (ESI): calcd for C_15_H_12_O_4_ (MH+): 257.0809; found 257.2691.

*(E)*-3-(3,4-Dimethoxyphenyl)-1-(3-hydroxyphenyl)prop-2-en-1-one (**18**)

Yellow power, 46.5% yield, mp 89.8~93.1 °C [115-116 °C, Lit^1^]. ^1^H-NMR (600 MHz, CDCl_3_) δ: 7.78 (d, *J* = 15.6 Hz, 1H), 7.61 (d, *J* = 1.8 Hz, 1H), 7.57 (d, *J* = 7.8 Hz, 1H), 7.38 (t, *J* = 7.8 Hz, 1H), 7.37 (d, *J* = 15.6 Hz, 1H), 7.23 (dd, *J_1_* = 1.8 Hz, *J_2_* = 8.4 Hz, 1H), 7.15 (d, *J* = 1.8 Hz, 1H), 7.11 (dd, *J_1_* =2.4 Hz, *J_2_* = 8.4 Hz, 1H), 6.89 (d, *J* = 8.4 Hz, 1H), 3.95 (s, 3H), 3.93 (s, 3H). ESI-MS m/z: 282.7 (M-H)^-^. HRMS (ESI): calcd for C_17_H_16_O_4_ (MH+): 285.1122; found 285.1137.

*(E)*-3-(3,4-Dihydroxyphenyl)-1-(2-fluorophenyl)prop-2-en-1-one (**19**)

Yellow green powder, 72.63% yield, mp 174.7~175.8 °C. ^1^H-NMR (600 MHz, Acetone-*d_6_*) δ: 8.58 (s, 1H), 8.26 (s, 1H), 7.76 (td, *J_1_* = 1.8 Hz, *J_2_* = 7.2 Hz, 1H), 7.63~7.64 (m, 1H), 7.57 (dd, *J_1_* = 1.2 Hz, *J_2_* = 15.6 Hz, 1H), 7.33~7.36 (m, 1H), 7.28~7.30 (m, 1H), 7.27 (d, *J* = 1.8 Hz, 1H), 7.21 (dd, *J_1_* = 2.4 Hz, *J_2_* = 15.6 Hz, 1H), 7.14 (dd, *J_1_* = 2.4 Hz, *J_2_* = 8.4 Hz, 1H), 6.90 (d, *J* = 7.8 Hz, 1H). ESI-MS m/z: 259.1 (M+H)^+^. HRMS (ESI): calcd for C_15_H_11_FO_3_ (MH+): 259.0765; found 259.0775.

*(E)*-1-(3,4-Dimethoxyphenyl)-3-(3,4,5-trimethoxyphenyl)prop-2-en-1-one (**20**)

Light yellow power, 83.3% yield, mp 123.9~125.6 °C Lit^6^, HPLC purity 99.7% (r.t. 8.927 min). ^1^H-NMR (600MHz, CDCl_3_) δ: 7.73 (d, *J* = 15.6 Hz, 1H), 7.69 (dd , *J_1_* = 1.8 Hz, *J_2_* = 8.4 Hz, 1H), 7.63 (d, *J* = 1.8 Hz, 1H), 7.43 (d, *J* = 15.6 Hz, 1H), 6.94 (d , *J* = 8.4 Hz, 1H), 6.87 (s, 2H), 3.98 (s, 6H), 3.93 (s, 6H), 3.91 (s, 3H). ^13^C-NMR (600 MHz, CDCl_3_): 188.11, 153.00, 152.80, 148.83, 143.66, 139.86, 130.90, 130.10, 122.52, 120.60, 110.39, 109.46, 105.17, 60.51, 55.80, 55.61. ESI-MS m/z: 359.3 (M+H)^+^. HRMS (ESI): calcd for C_20_H_22_O_6_ (MH+): 359.1489; found 359.1490.

*(E)*-3-(3,4-Dimethoxyphenyl)-1-(4-hydroxyphenyl)prop-2-en-1-one (**21**)

Orange yellow power, 73.51% yield, mp 175.7~178.7 °C. ^1^H-NMR (600 MHz, Acetone-*d_6_*) δ: 9.15 (s, 1H), 8.05 (dd, *J_1_* = 8.4 Hz, *J_2_* = 7.2 Hz, 2H), 7.74 (d, *J* = 15.6 Hz, 1H), 7.69 (d, *J* = 15.6 Hz, 1H), 7.48 (d, *J* = 2.4 Hz, 1H), 7.32 (dd, *J_1_* = 2.4 Hz, *J_2_* = 8.4 Hz, 1H), 7.01 (d, *J* = 8.4 Hz, 1H), 6.96 (dd, *J_1_* = 1.8 Hz, *J_2_* = 6.6 Hz, 2H), 3.89 (s, 3H), 3.86 (s, 3H). ESI-MS m/z: 285.1 (M+H)^+^. HRMS (ESI): calcd for C_17_H_16_O_4_ (MH+): 285.1122; found 285.1125.

*(E)*-3-(3,4-Dichlorophenyl)-1-(3,4-dimethoxyphenyl)prop-2-en-1-one (**22**)

Yellowish powder, 8.84% yield, mp 99.4~101.5 °C. ^1^H-NMR (600 MHz, CDCl_3_) δ: 7.73 ( d, *J* = 1.8 Hz, 1H), 7.69 (d, *J* = 16.2 Hz, 1H), 7.68 (dd, *J_1_* = 1.8 Hz, *J_2_* = 8.4 Hz, 1H), 7.62 (d, *J* = 1.8 Hz, 1H), 7.53 (d, *J* = 15.6 Hz, 1H), 7.50 (d, *J* = 8.4 Hz, 1H), 7.45 (dd, *J_1_* = 1.8 Hz, *J_2_* = 8.4 Hz, 1H), 6.94 (d, *J* = 8.4 Hz, 1H). ESI-MS m/z: 337.2 (M+H)^+^. HRMS (ESI): calcd for C_17_H_14_Cl_2_O_3_ (MH+): 337.0393; found 337.0393.

*(E)*-3-(2-Chlorophenyl)-1-(3,4-dihydroxyphenyl)prop-2-en-1-one (**23**)

Dark purple syrupy, 43.5% yield. ^1^H-NMR (600 MHz, Acetone-*d_6_*) δ: 8.13 (d, *J* = 15.6 Hz, 1H), 8.10 (dd, *J_1_* = 1.8 Hz, *J_2_* = 8.4 Hz, 1H), 7.85 (d, *J* = 15.6 Hz, 1H), 7.69 (dd, *J_1_* = 2.4 Hz, *J_2_* = 8.4 Hz, 1H), 7.66 (d, *J* = 1.8 Hz, 1H), 7.53 (dd, *J_1_* = 1.8 Hz, *J_2_* = 7.8 Hz, 1H), 7.43~7.45 (m, 2H). ESI-MS m/z: 272.8 (M-H)^-^. HRMS (ESI): calcd for C_15_H_11_ClO_3_ (MH+): 275.0470; found 275.0468.

*(E)*-1-(3,4-Dihydroxyphenyl)-3-(2,3-dimethoxyphenyl)prop-2-en-1-one (**24**)

Brown yellow powder, 20.03% yield, mp 160.2~163.2 °C, HPLC purity 95.9% (r.t. 8.789 min). ^1^H-NMR (500 MHz, Acetone-*d_6_*) δ: 8.53 (s, 2H), 7.90 (d, *J* = 16.0 Hz, 1H), 7.81 (d, *J* = 15.5 Hz, 1H), 7.62 (d, *J* = 8.5 Hz, 1H), 7.58 (t, *J* = 9.5 Hz, 1H), 7.52 (s, 1H), 7.14~7.15 (m, 2H), 6.87 (d, *J* = 8.5 Hz, 1H), 3.84 (s, 3H), 3.79 (s, 3H). ^13^C-NMR (600 MHz, DMSO-*d_6_*): 187.26, 152.77, 150.93, 148.07, 145.50, 136.48, 129.58, 128.44, 124.28, 123.04, 122.07, 119.12, 115.33, 115.10, 114.65, 60.88, 55.80. ESI-MS m/z: 300.9 (M+H)^+^. HRMS (ESI): calcd for C_17_H_16_O_5_ (MH+): 301.1071; found 301.1075.

*(E)*-1-(3,4-Dihydroxyphenyl)-3-(3,4-dimethoxyphenyl)prop-2-en-1-one (**25**)

Yellow powder, 64.34% yield, mp 109.5~111.0 °C, HPLC purity 100.0% (r.t. 7.593 min). ^1^H-NMR (500 MHz, Acetone-*d_6_*) δ: 7.71 (d, *J* = 15.6 Hz, 1H), 7.67 (d, *J* = 15.6 Hz, 1H), 7.62~7.63 (m, 2H), 7.48 (d, *J* = 1.8 Hz, 1H), 7.32 (dd, *J_1_* = 1.8 Hz, *J_2_* = 7.8 Hz, 1H), 7.01 (d, *J* = 8.4 Hz, 1H), 7.94 (d, *J* = 9.0 Hz, 1H), 3.90 (s, 3H), 3.87 (s, 3H). ESI-MS m/z: 301.5 (M+H)^+^. HRMS (ESI): calcd for C_17_H_16_O_5_ (MH+): 301.1071; found 301.1071.

*(E)*-1-(3,4-Dihydroxyphenyl)-3-(3-hydroxyphenyl)prop-2-en-1-one (**26**)

Brown red powder, 8.15% yield, mp 218.5~219.9 °C. ^1^H-NMR (500 MHz, Acetone-*d_6_*) δ: 9.95 (s, 1H), 9.60 (s, 1H), 9.38 (s, 1H), 7.73 (d, *J* = 16.0 Hz, 1H), 7.62 (dd, *J_1_* = 2.0 Hz, *J_2_* = 8.5 Hz, 1H), 7.55 (d, *J* = 15.5 Hz, 1H), 7.50 (d, *J* = 2.0 Hz, 1H), 7.264 (t, *J* = 8.5 Hz, 1H), 7.24 (d, *J* = 7.5 Hz, 1H), 7.18 (s, 1H), 6.86 (d, *J* = 9.0 Hz, 1H), 6.85 (d, *J* = 9.0 Hz, 1H). ^13^C-NMR (600 MHz, DMSO-*d_6_*): 187.14, 157.70, 151.19, 145.56, 136.19, 129.84, 129.41, 122.10, 122.00, 119.53, 117.40, 115.24, 115.09, 115.00. ESI-MS m/z: 254.7 (M-H)^-^. HRMS (ESI): calcd for C_15_H_12_O_4_ (MH+): 257.0809; found 257.0810.

*(E)*-1-(3,4-Dihydroxyphenyl)-3-(4-methoxyphenyl)prop-2-en-1-one (**27**)

Yellow crystal, 81.62% yield, mp 129.3~132.3 °C. ^1^H-NMR (600 MHz, CDCl_3_) δ: 7.80 (d, *J* = 9.0 Hz, 2H), 7.70 (d, *J* = 15.0 Hz, 1H), 7.61 (d, *J* = 15.0 Hz, 1H), 7.61 (dd, *J_1_* = 2.4 Hz, *J_2_* = 8.4 Hz, 1H), 7.50 (d, *J* = 2.4 Hz, 1H), 7.00 (d, *J* = 9.0 Hz, 2H), 6.85 (d, *J* = 8.4 Hz, 1H), 3.81 (s, 3H). ESI-MS m/z: 268.8 (M-H)^-^. HRMS (ESI): calcd for C_16_H_14_O_4_ (MH+): 271.0963; found 271.0965.

*(E)*-1-(3,4-Dihydroxyphenyl)-3-(4-hydroxyphenyl)prop-2-en-1-one (**28**)

Brown red powder, 11.61% yield, mp 133.1~135 °C. ^1^H-NMR (500 MHz, DMSO-*d_6_*) δ: 10.00 (s, 1H), 9.85 (brs, 1H), 9.31 (brs, 1H), 7.68 (d, *J* = 9.0 Hz, 2H), 7.62 (d, *J* = 15.6 Hz, 1H), 7.57 (d, *J* = 15.6 Hz, 1H), 7.58 (dd, *J_1_* = 1.8 Hz, *J_2_* = 7.8 Hz, 1H), 7.48 (d, *J* = 1.8 Hz, 1H), 7.84 (d, *J* = 7.8 Hz, 1H), 7.82 (d, *J* = 9.0 Hz, 2H). ESI-MS m/z: 254.8 (M-H)^-^. HRMS (ESI): calcd for C_15_H_12_O_4_ (MH+): 257.0809; found 257.0809.

*(E)*-1-(3,4-Dihydroxyphenyl)-3-(2-methoxyphenyl)prop-2-en-1-one (**29**)

Red-brown power, 50.82% yield, mp 145.2~147.9 °C, HPLC purity 96.9% (r.t. 9.437 min). ^1^H-NMR (600 MHz, CDCl_3_) δ: 8.11 (d, *J* = 16.2 Hz, 1H), 7.64 (d, *J* = 16.2 Hz, 1H), 7.77 (s, 1H), 7.59~7.64 (m, 2H), 7.38 (t, *J* = 7.8 Hz, 1H), 6.94~7.01 (m, 3H), 3.92 (s, 3H). ^13^C-NMR (600 MHz, DMSO-*d_6_*): 187.33, 158.10, 150.80, 145.46, 137.04, 131.84, 129.70, 128.40, 123.19, 122.21, 121.93, 120.67, 115.32, 115.10, 111.74, 55.68. ESI-MS m/z: 268.8 (M-H)^-^. HRMS (ESI): calcd for C_16_H_14_O_4_ (MH+): 271.0965; found 271.0973.

*(E)*-3-(3,4-Dichlorophenyl)-1-(3,4-dihydroxyphenyl)prop-2-en-1-one (**30**)

Dark purple syrupy, 49.21% yield. ^1^H-NMR (600 MHz, Acetone-*d_6_*) δ: 8.09 (d, *J* = 1.8 Hz, 1H), 7.93 (d, *J* = 16.2 Hz, 1H), 7.79 (dd, *J_1_* = 1.8 Hz, *J_2_* = 8.4 Hz, 1H, H-6), 7.68 (d, *J* = 8.4 Hz, 1H), 7.66 (d, *J* = 16.2 Hz, 1H), 7.65 (d, *J* = 1.8 Hz, 1H), 7.44 (dd, *J_1_* = 1.8 Hz, *J_2_* = 8.4 Hz, 1H), 6.96 (d, *J* = 8.4 Hz, 1H). ESI-MS m/z: 308.7 (M+H)^+^. HRMS (ESI): calcd for C_15_H_10_Cl_2_O_3_ (MNa+): 330.9905; found 330.9909.

*(E)*-1-(3,4-Dihydroxyphenyl)-3-(3-fluorophenyl)prop-2-en-1-one (**31**)

Light yellow powder, 4.11% yield, mp 178.2~181.7, HPLC purity 95.0% (r.t. 8.313 min). ^1^H-NMR (500 MHz, Acetone-*d_6_*) δ: 10.0 (s, 1H), 9.39 (s, 1H), 7.93 (d, *J* = 15.5 Hz, 1H), 7.83 (d, *J* = 10.5 Hz, 1H), 7.65-7.69 (m, 2H), 7.64 (d, *J* = 16.0 Hz, 1H), 7.53 (s, 1H), 7.49-7.51 (m, 1H), 7.25-7.29 (m, 1H), 6.87 (d, *J* = 8.5 Hz, 1H). HRMS (ESI): calcd for C_15_H_11_FO_3_ (MH+): 259.0765; found 259.0765.

**3. The chemical structures of synthesized chalcone derivatives and their inhibitory effects on TNF-α and IL-6 production**

Chalcone derivatives were synthesized by aldol condensation in NaOH or HCl solution. Mouse peritoneal macrophages (MPMs) were obtained from ICR mice and cultured in RPMI-1640 medium at 37 ^o^C in a humidified atmosphere of 95% air and 5% CO_2_. After plating and 24 h of growth, MPMs were treated with 0.5 μg/mL LPS in the presence of vehicle or synthesized chalcones at the dose of 10 μM for another 22 h. Culture medium and protein samples were then collected. The TNF-α and IL-6 levels in the medium were determined by ELISA and normalized to the total amount of protein in the viable cell pellets.

**
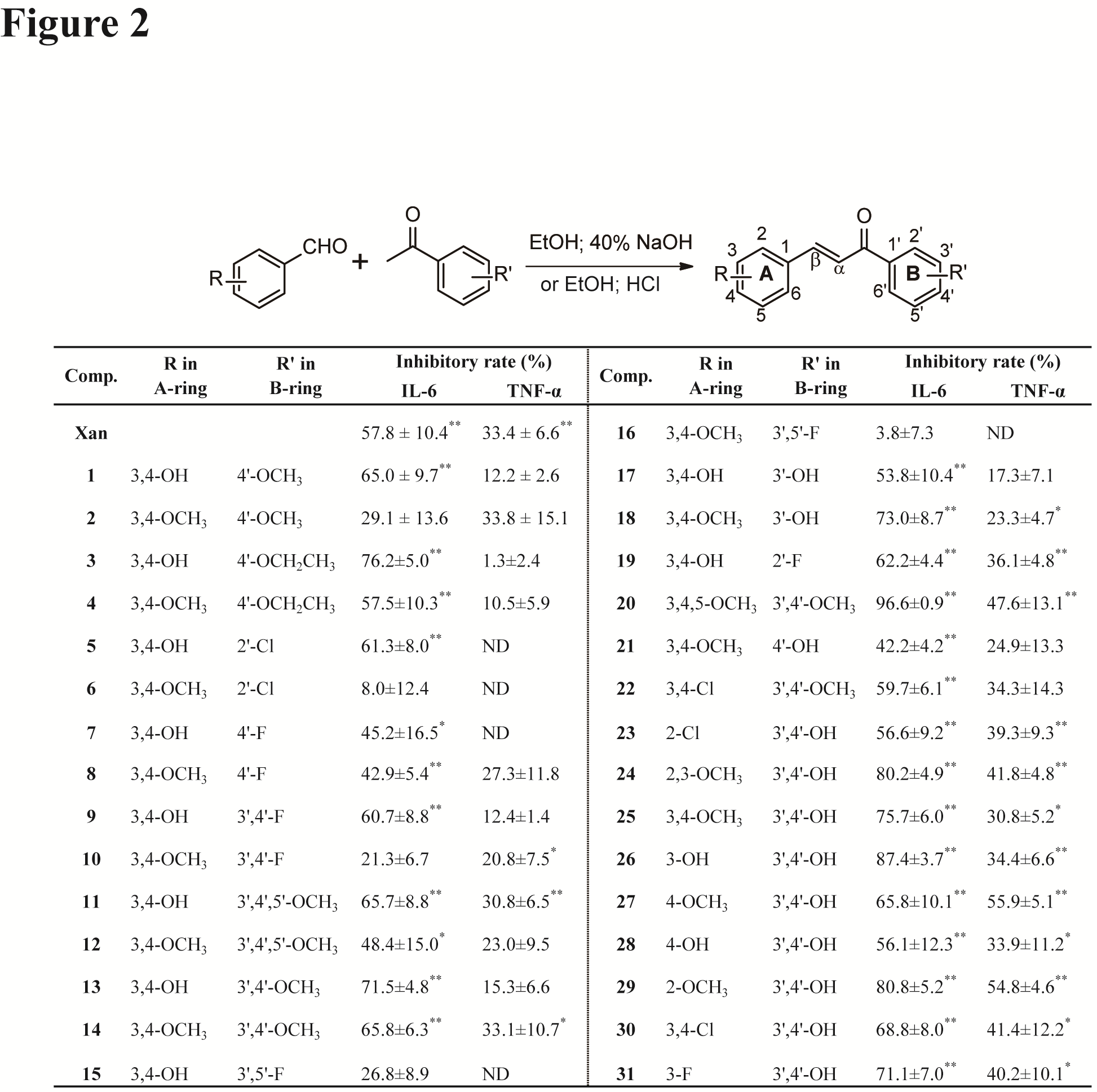
**

**Supplementary Figure S2.** The chemical structures of synthesized chalcone derivatives and their inhibitory effects on TNF-α and IL-6 production. Chalcone derivatives were synthesized by aldol condensation in NaOH or HCl solution. Data are expressed as inhibitory rate compared to LPS alone treated values, mean ± SEM. n ≥ 3. *p < 0.05 and **p < 0.01 vs. only-LPS stimulated group. ND, means the inhibitory rate was not detected.

**4. The original ^1^H-NMR spectra of compounds 1-31**


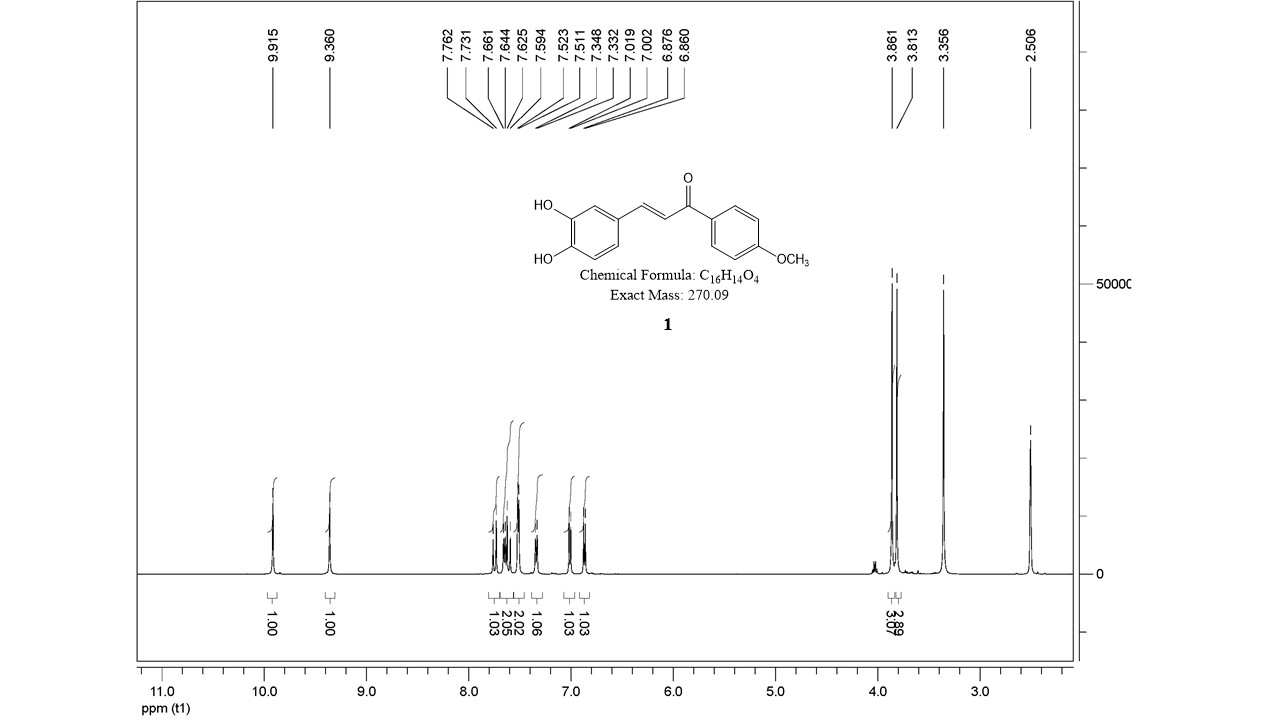

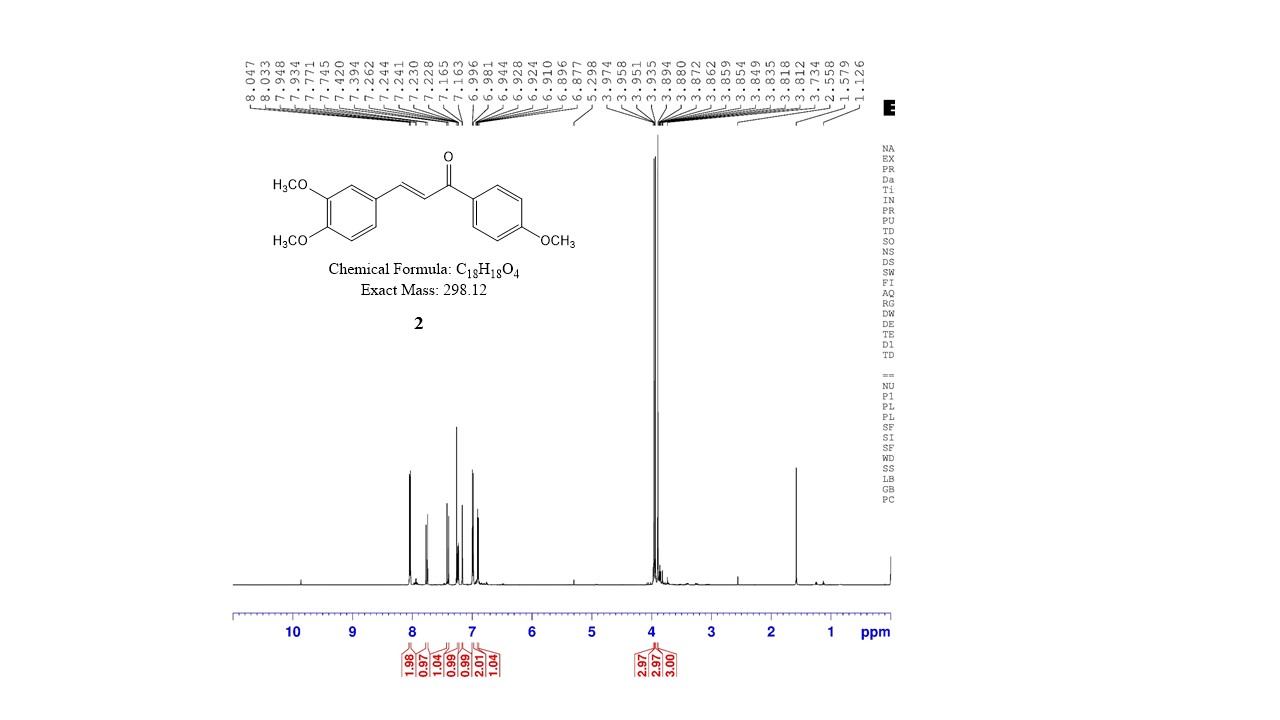


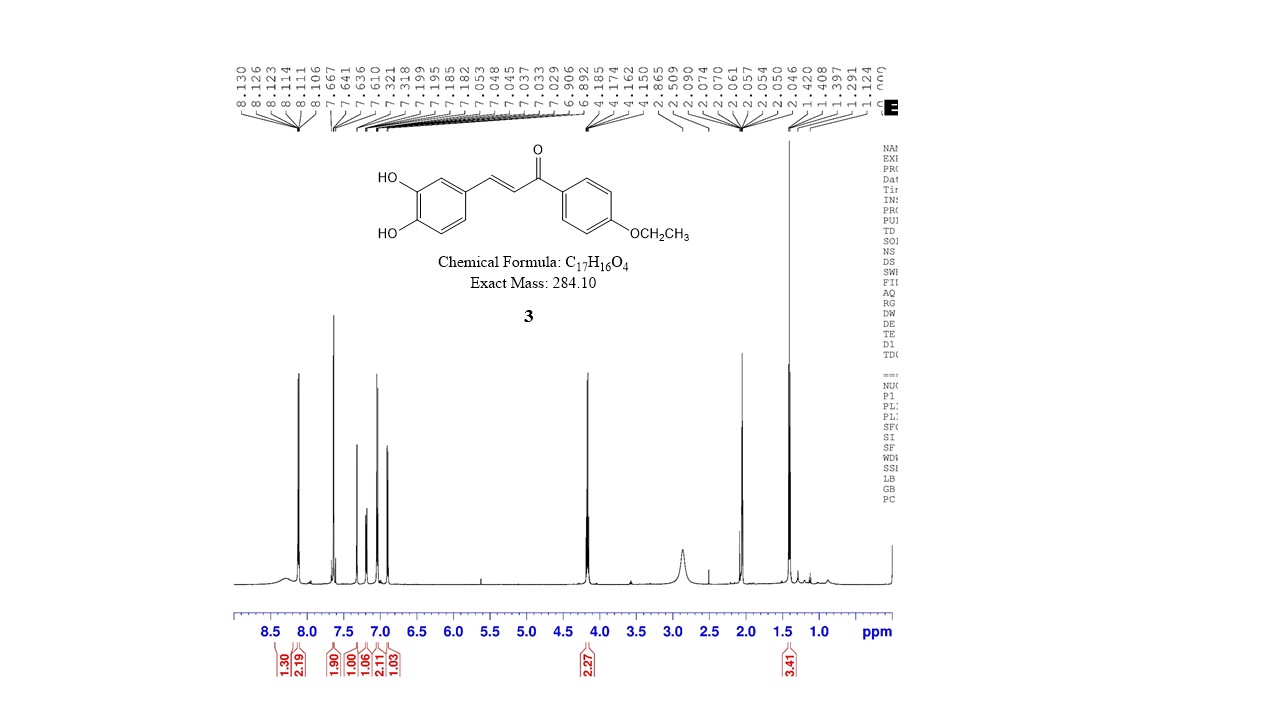

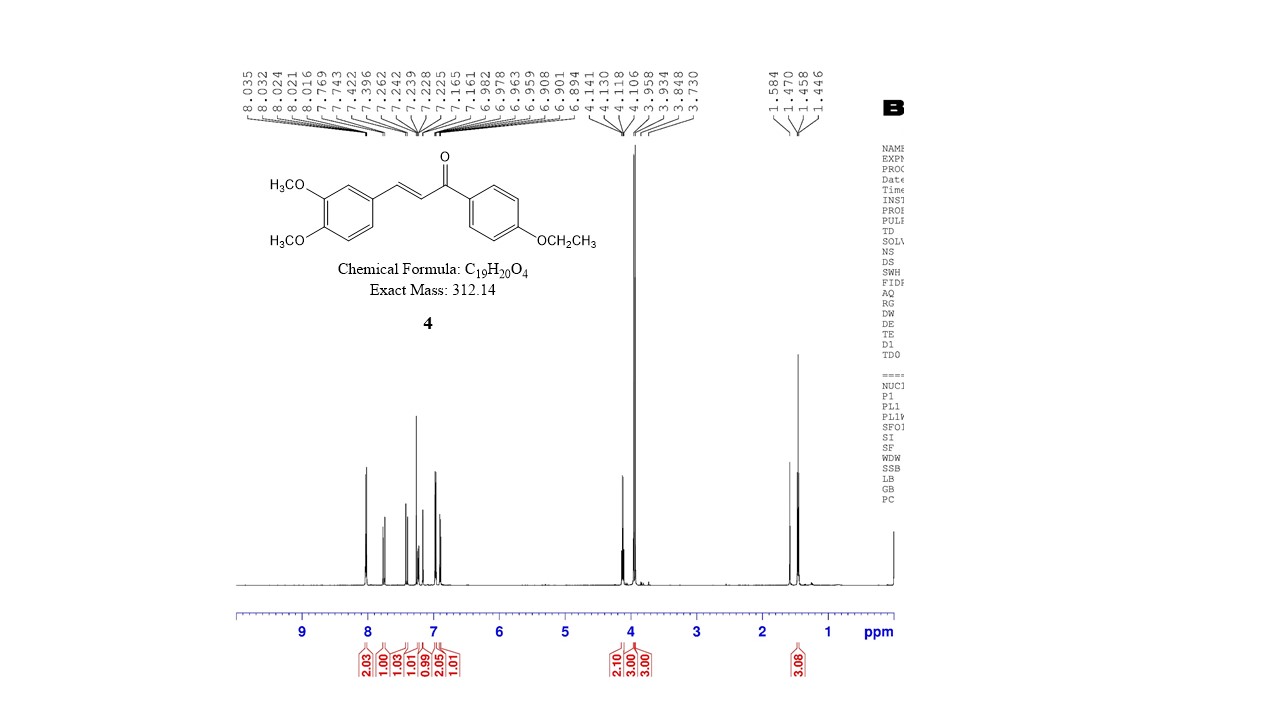


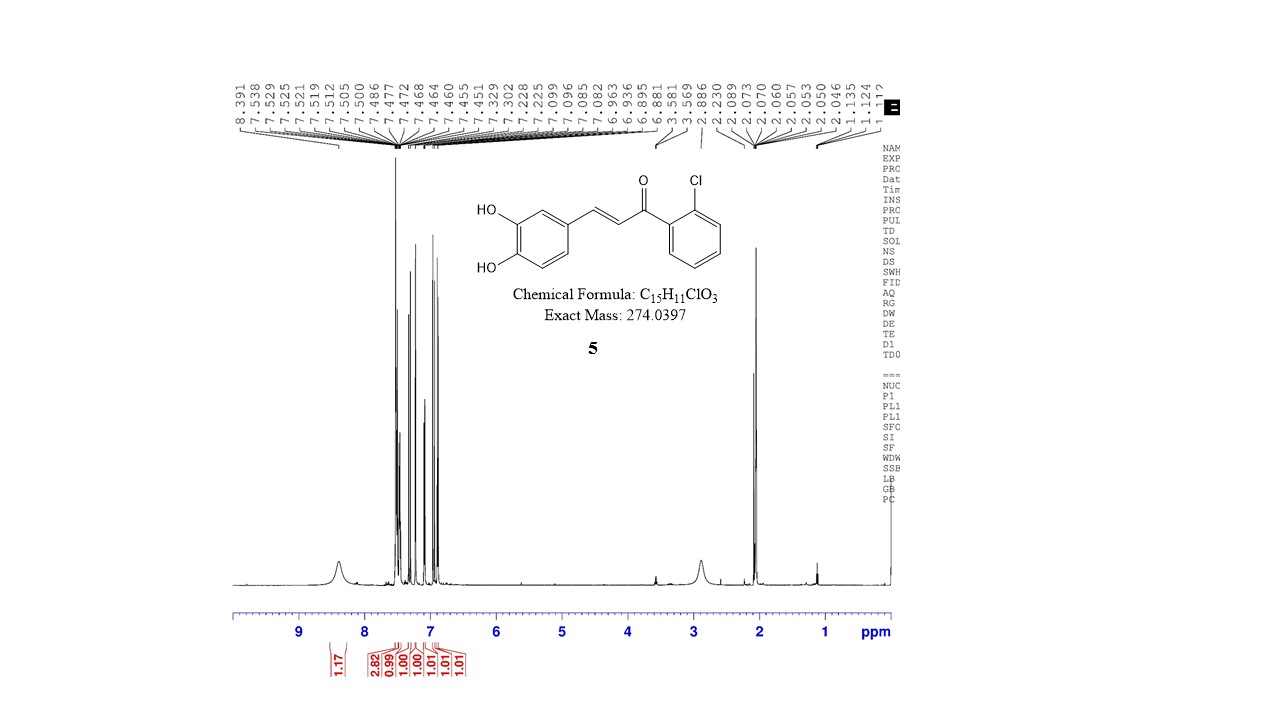

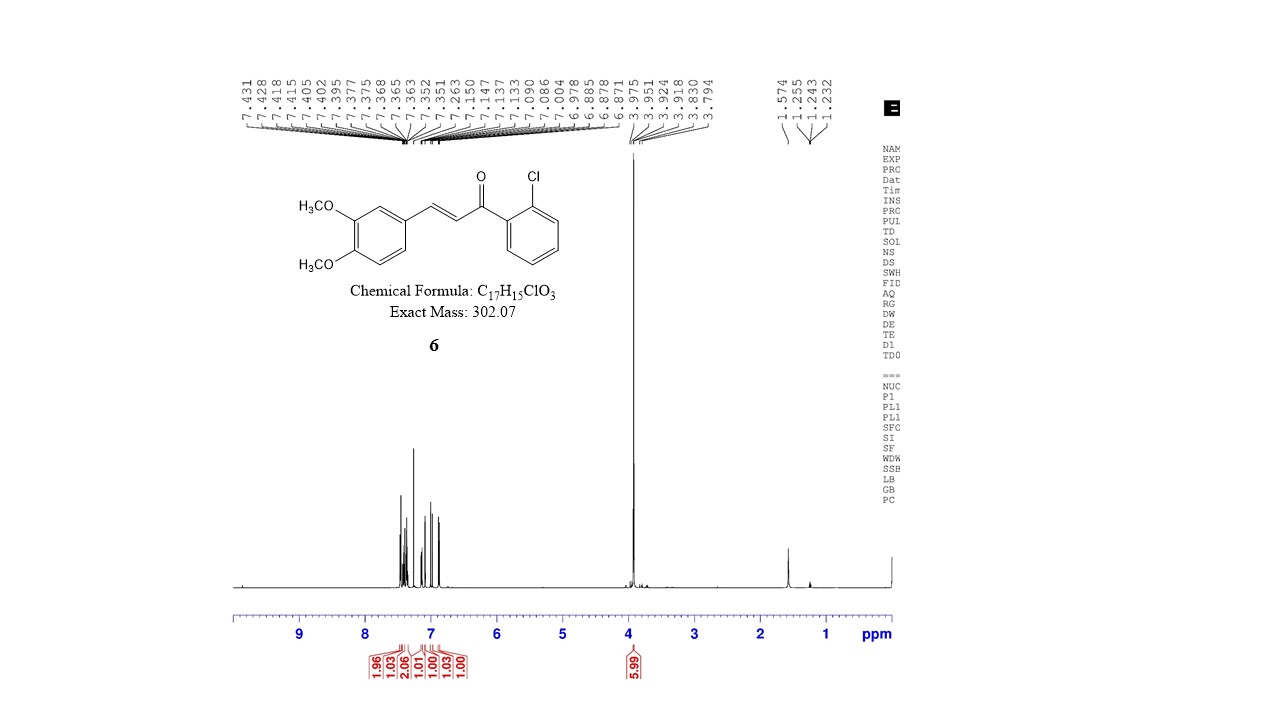


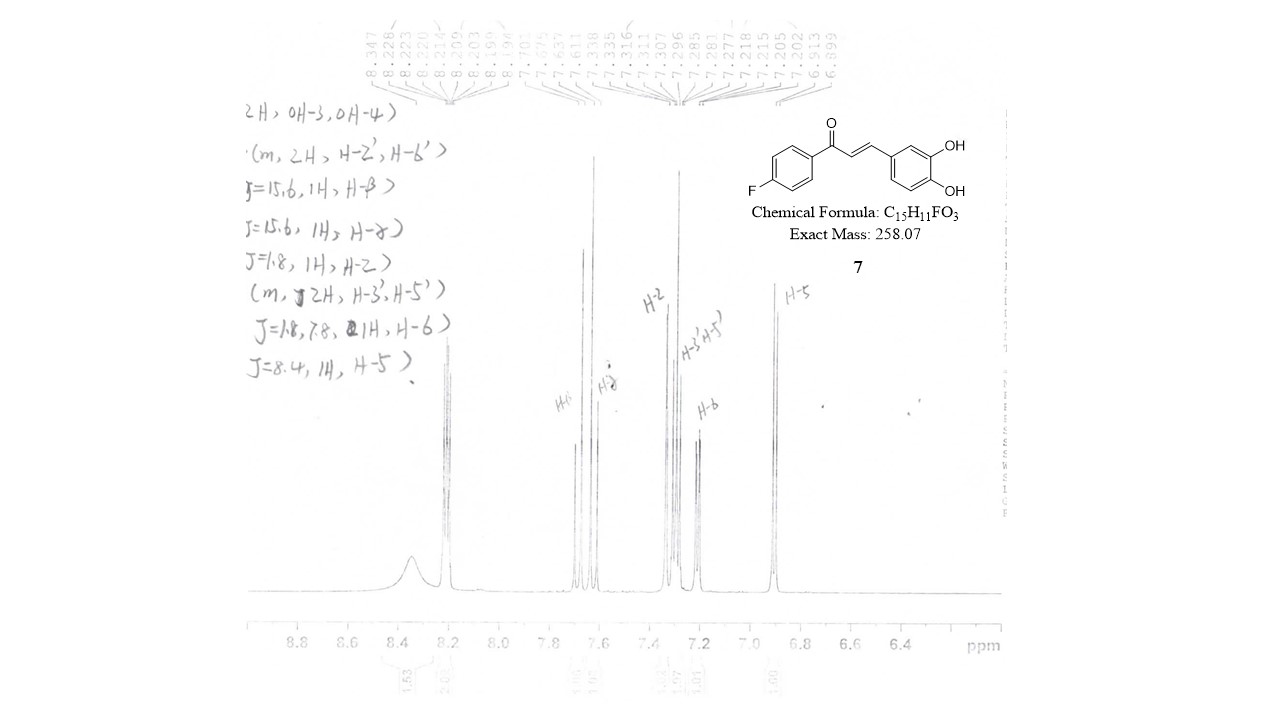

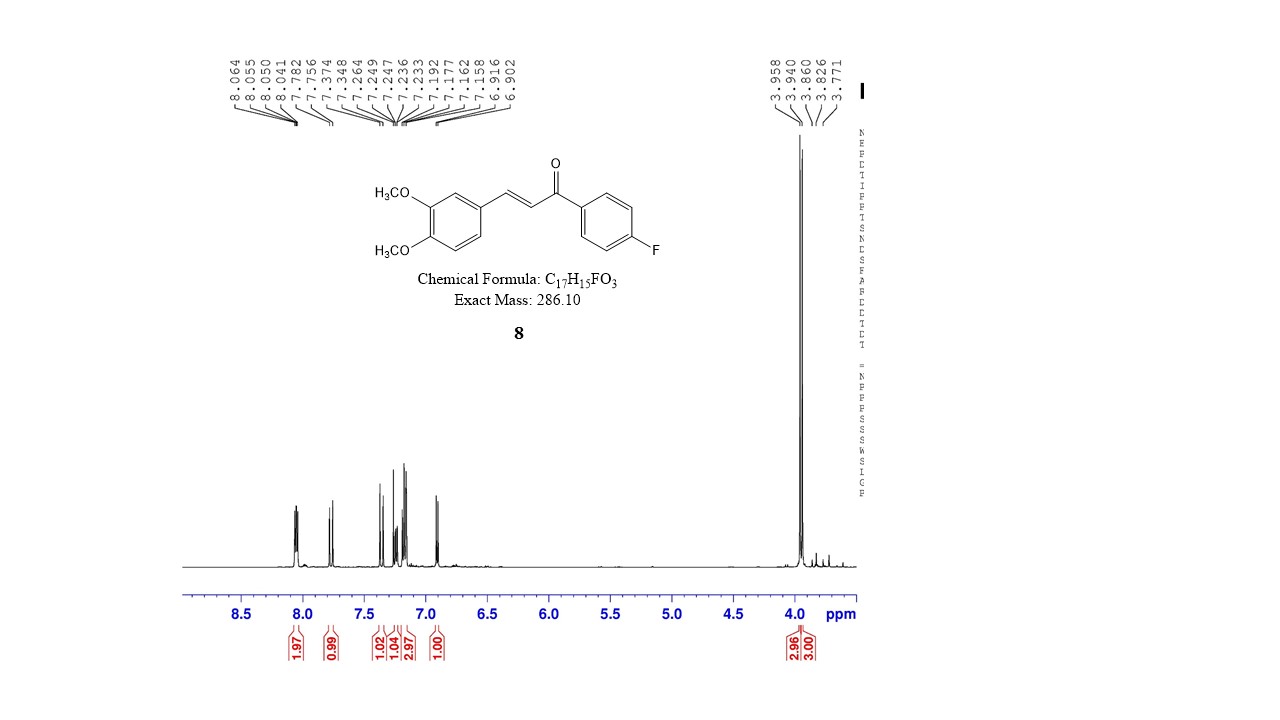


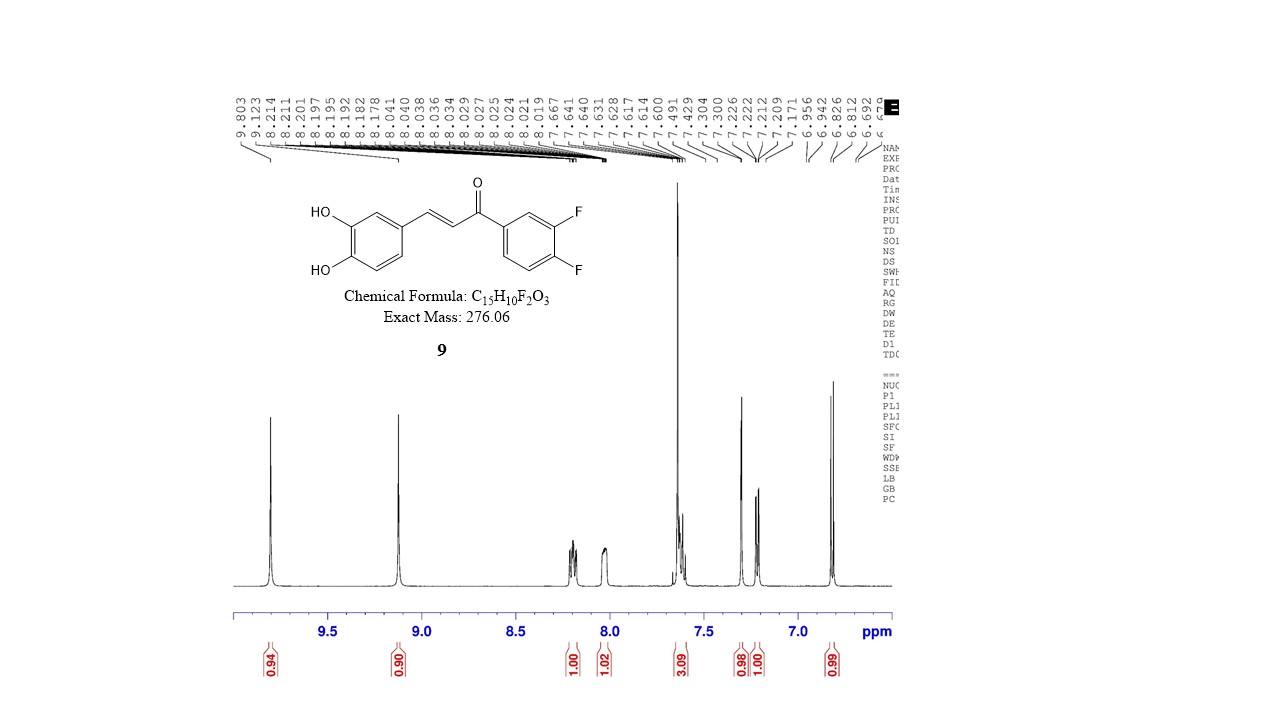

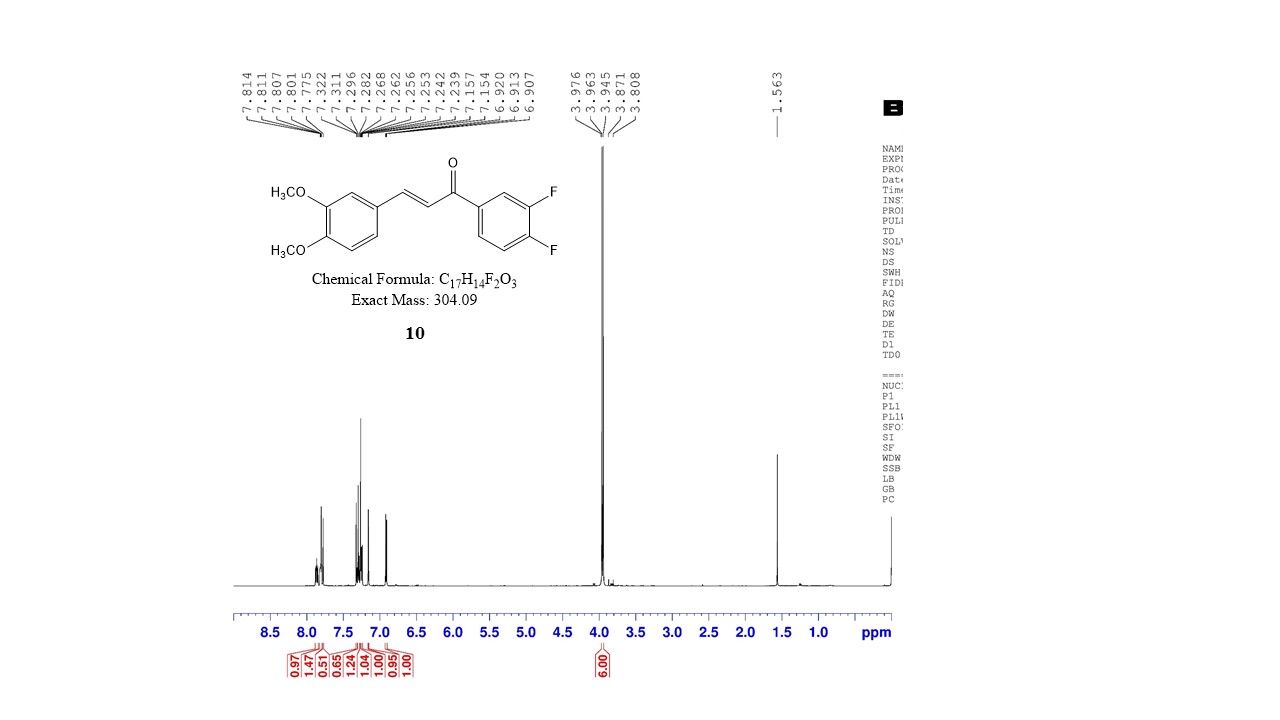


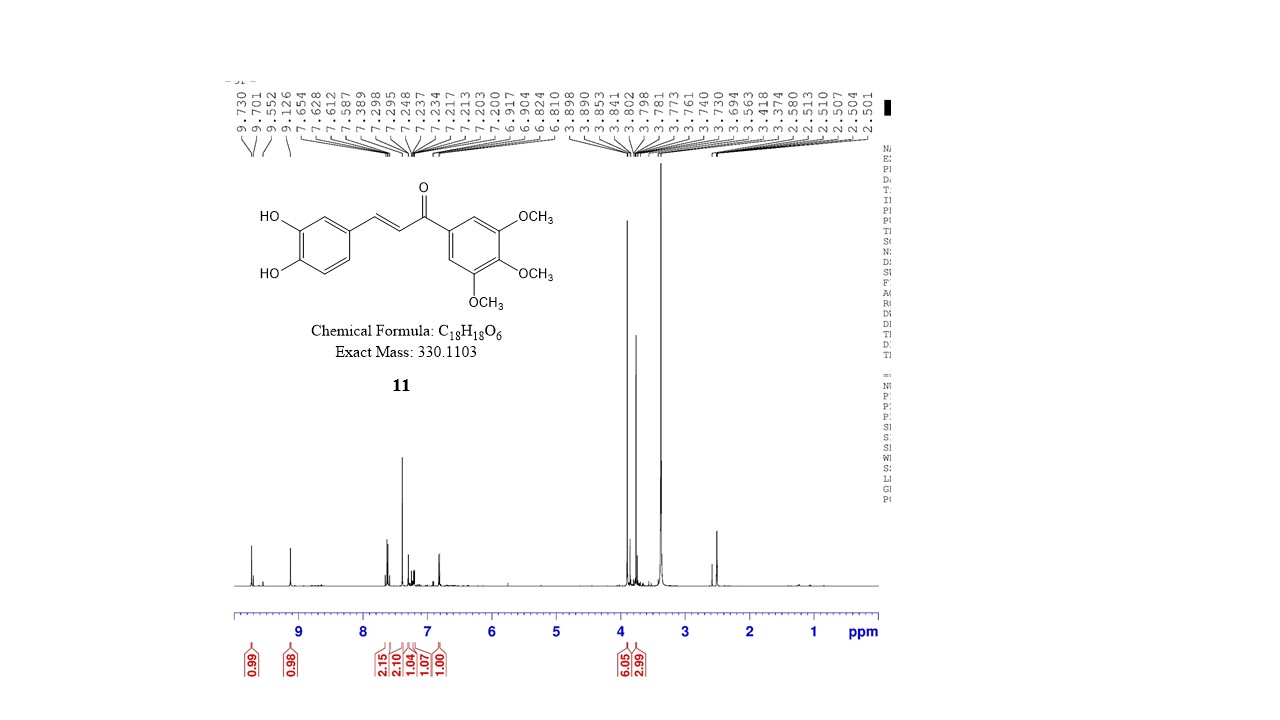

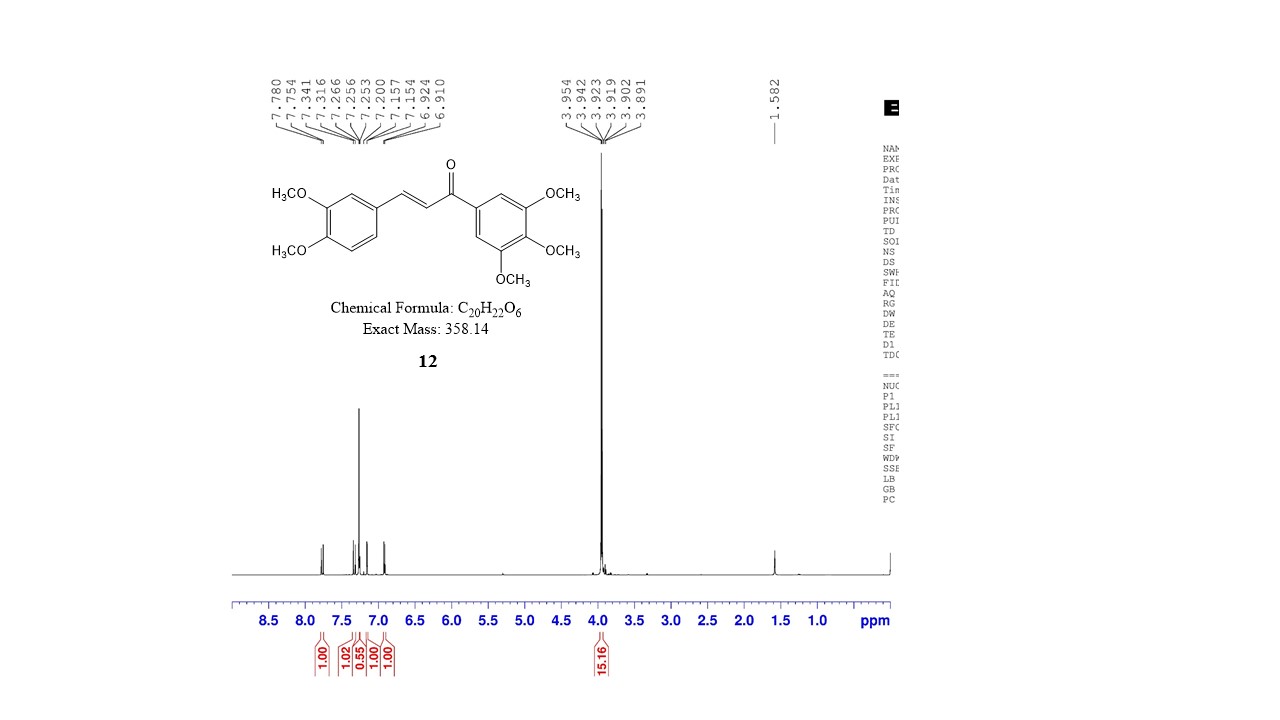


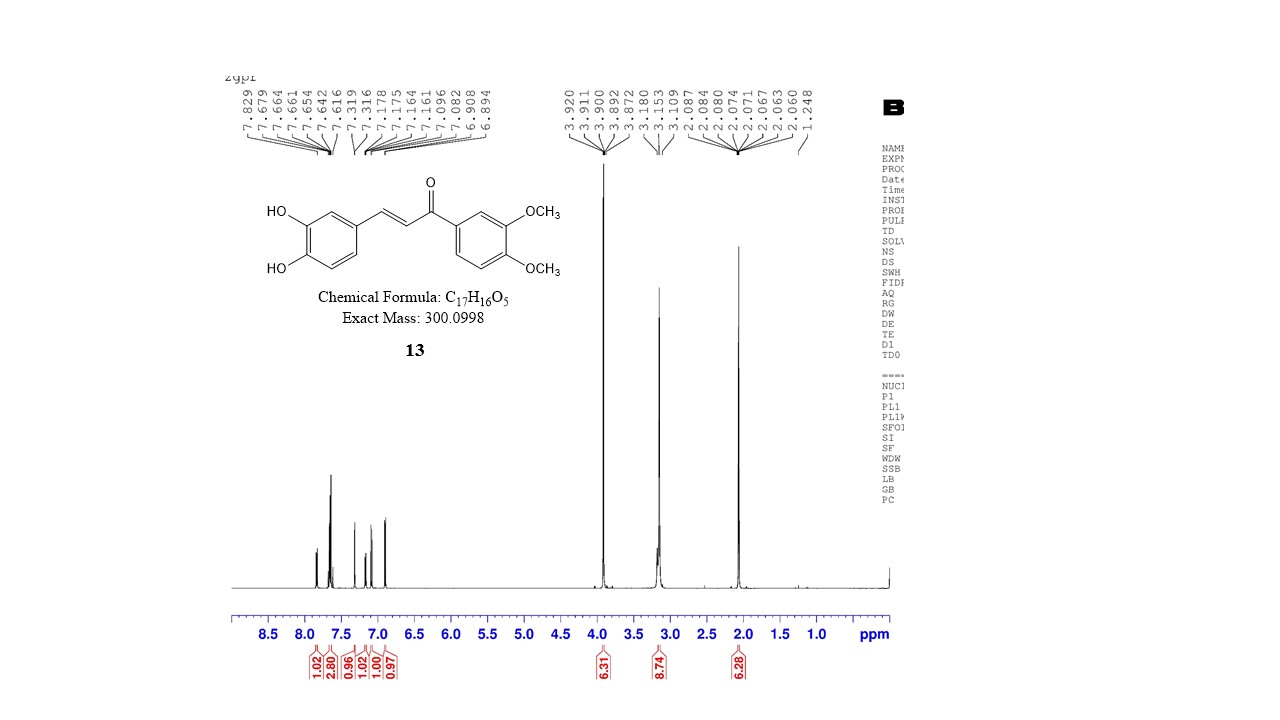

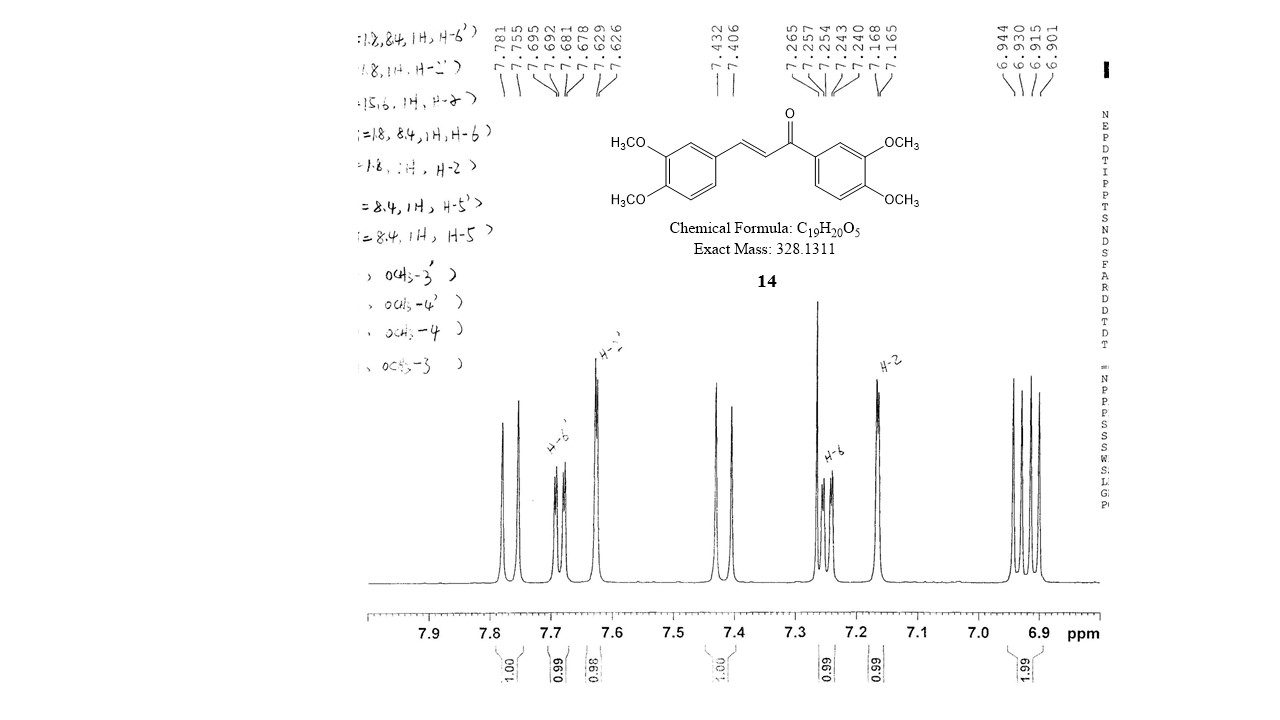


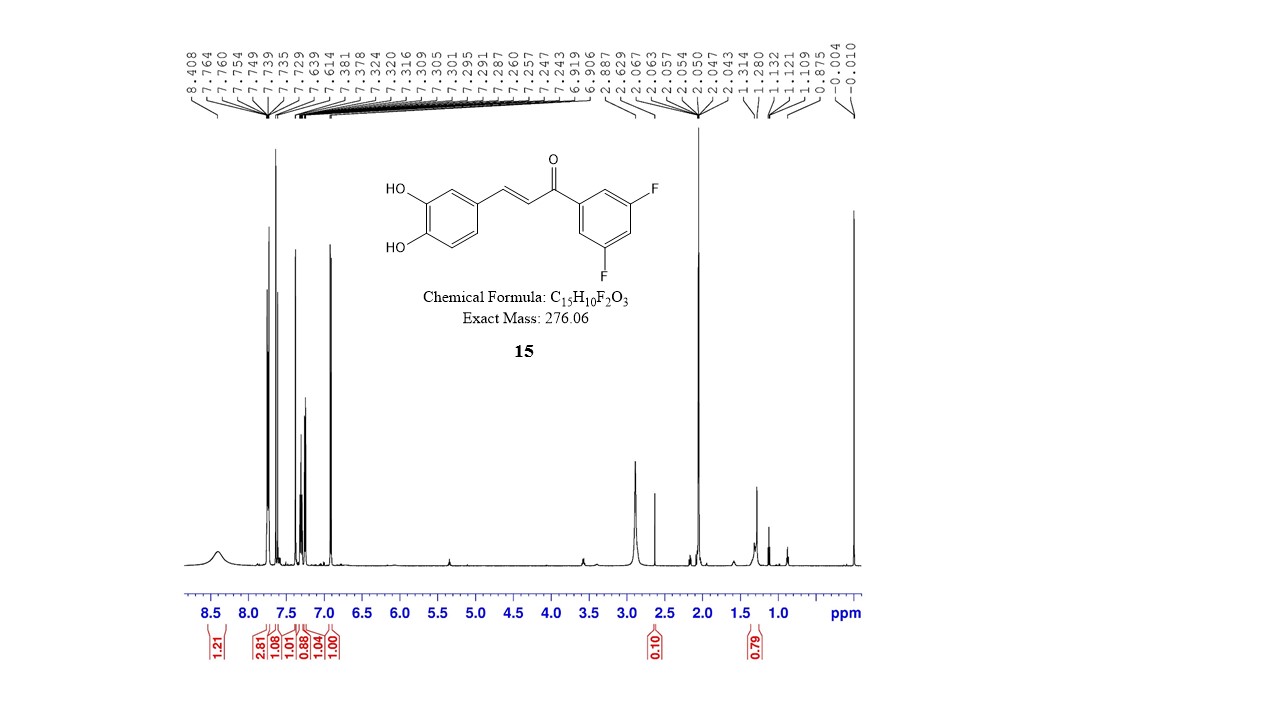

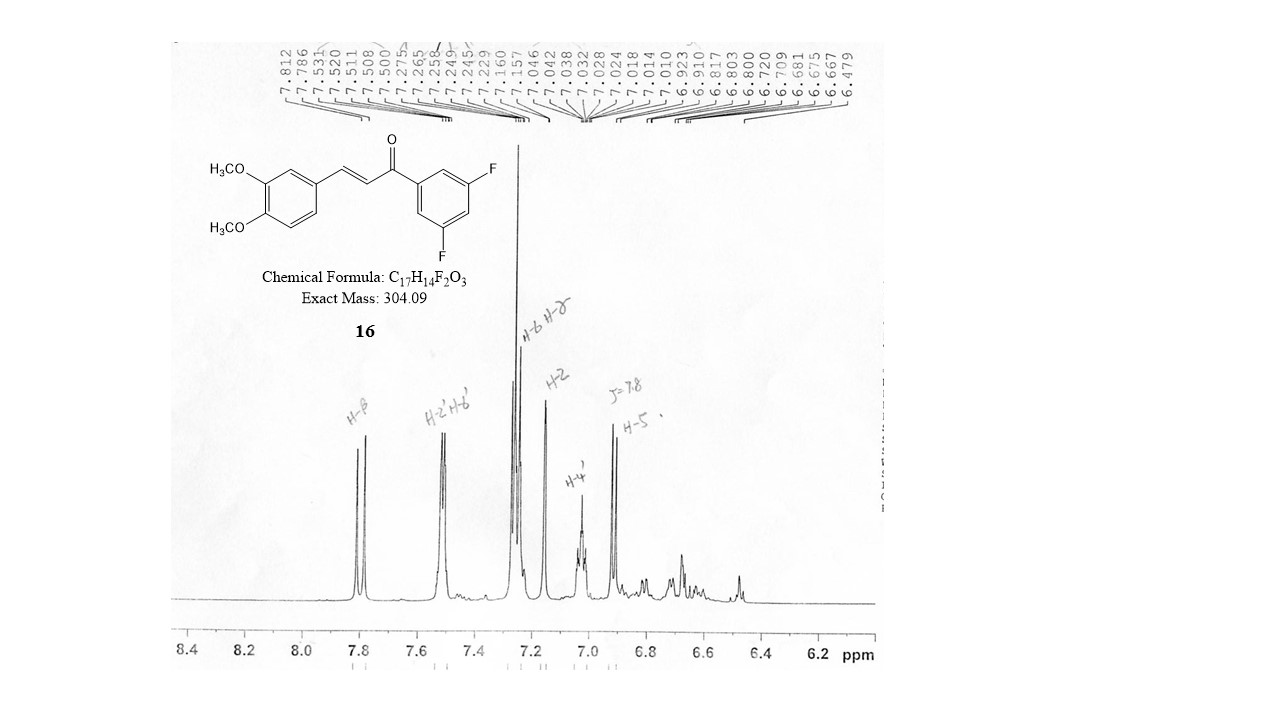


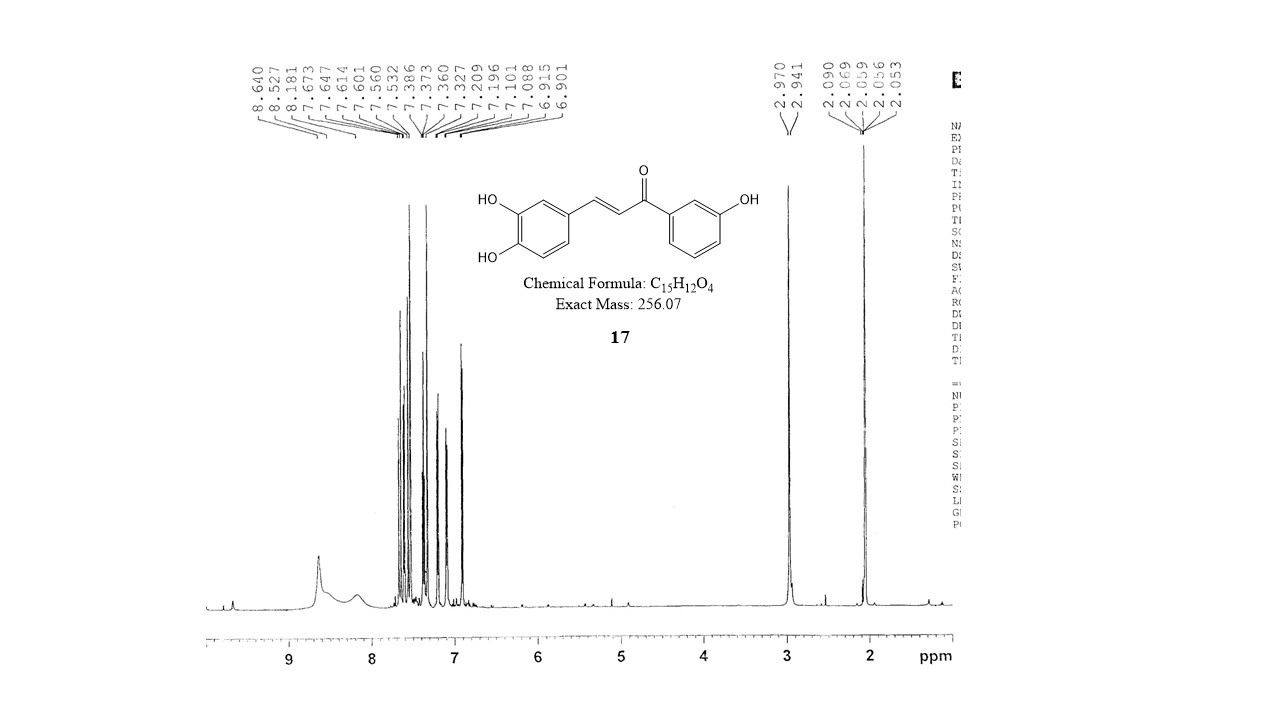

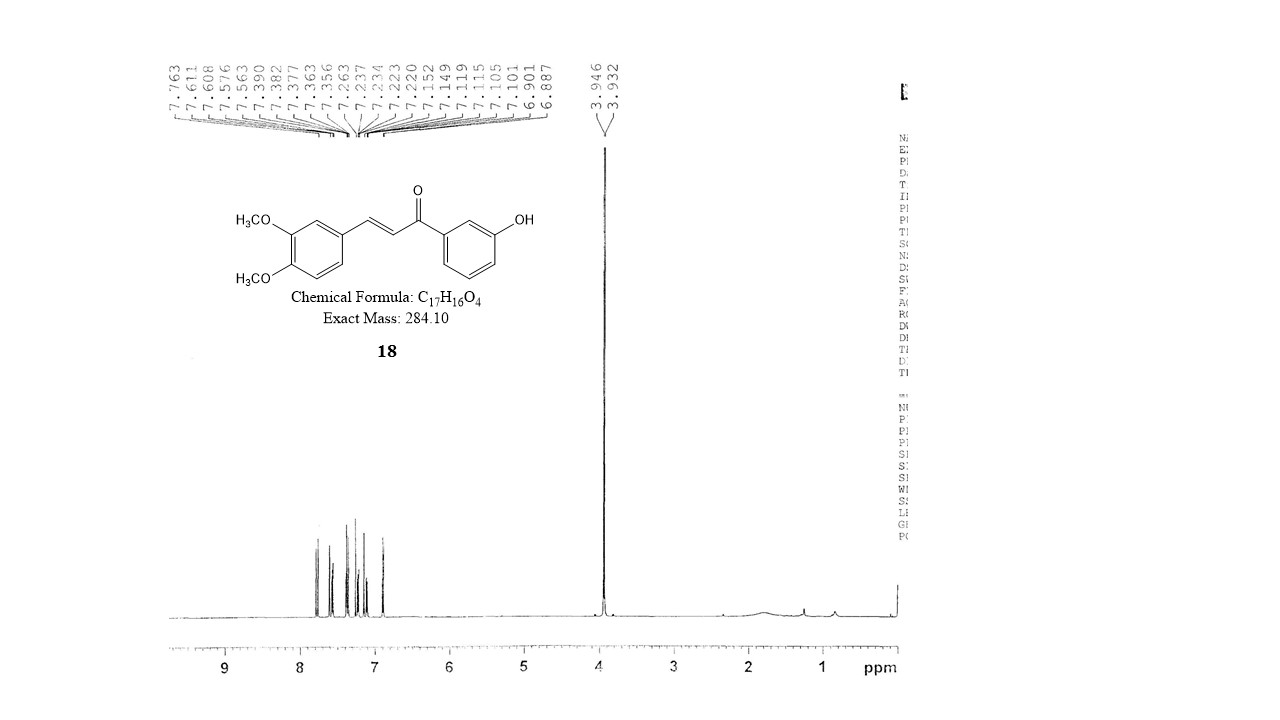


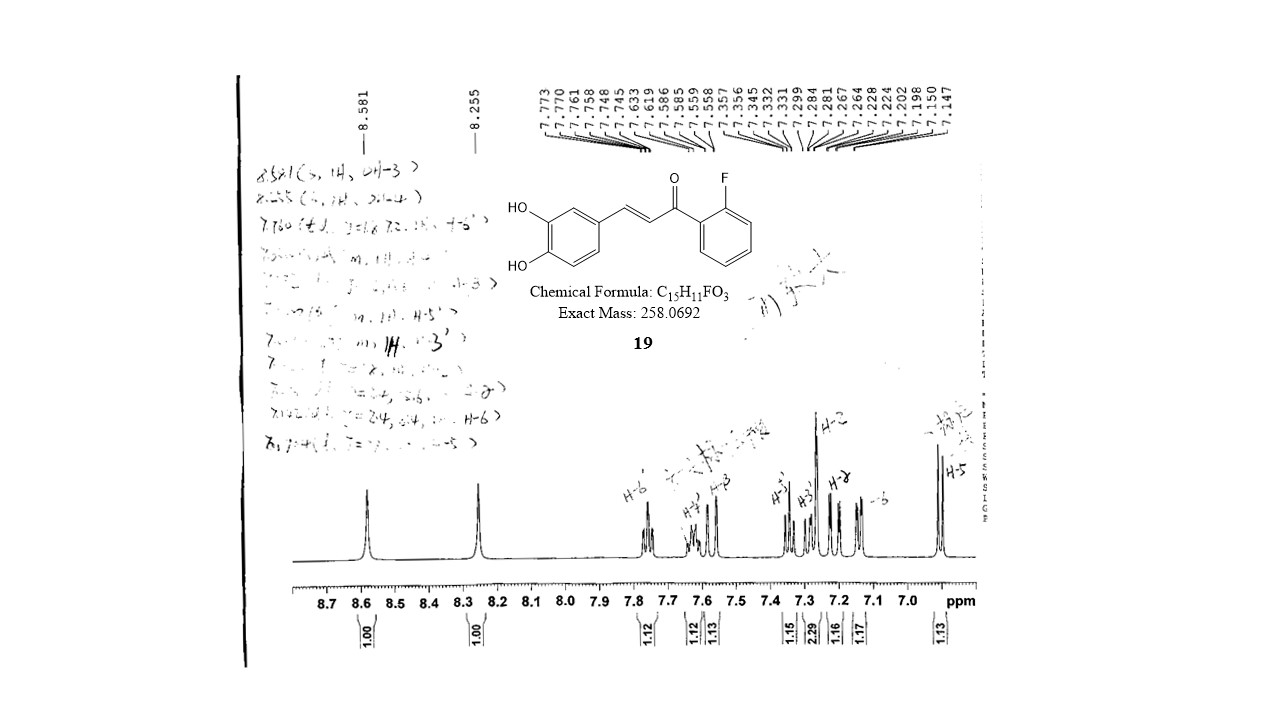

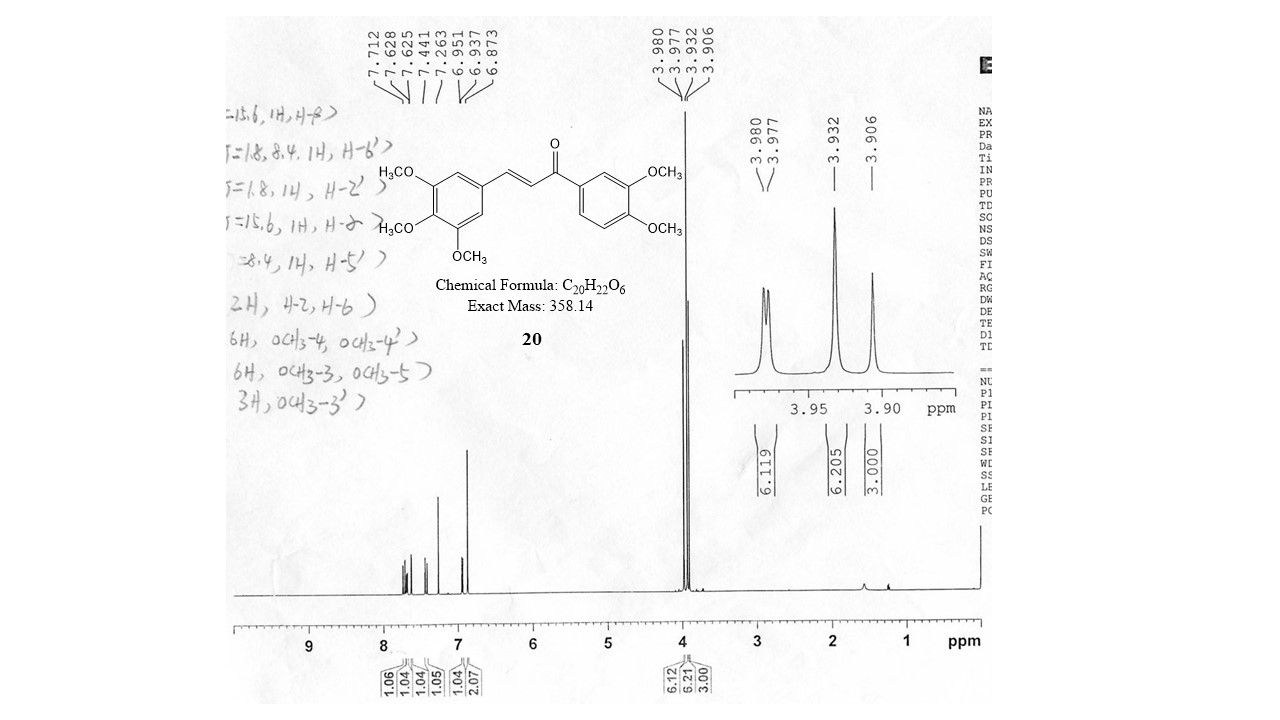


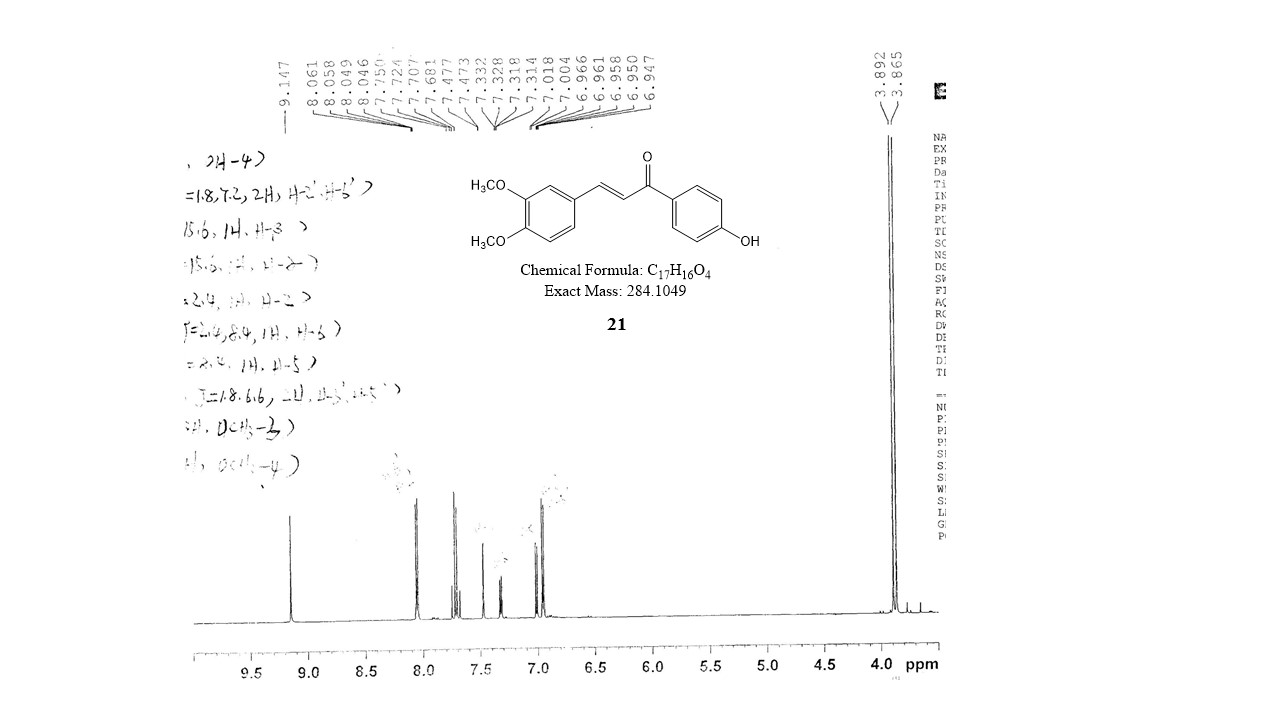

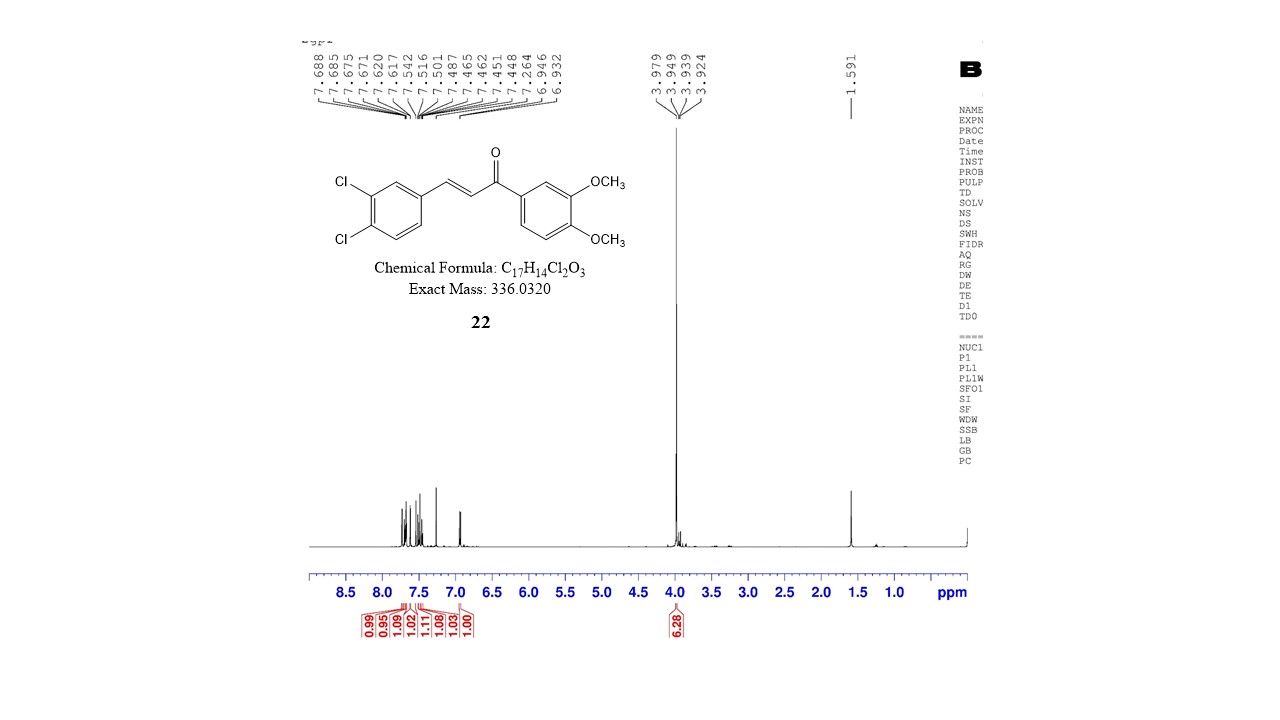


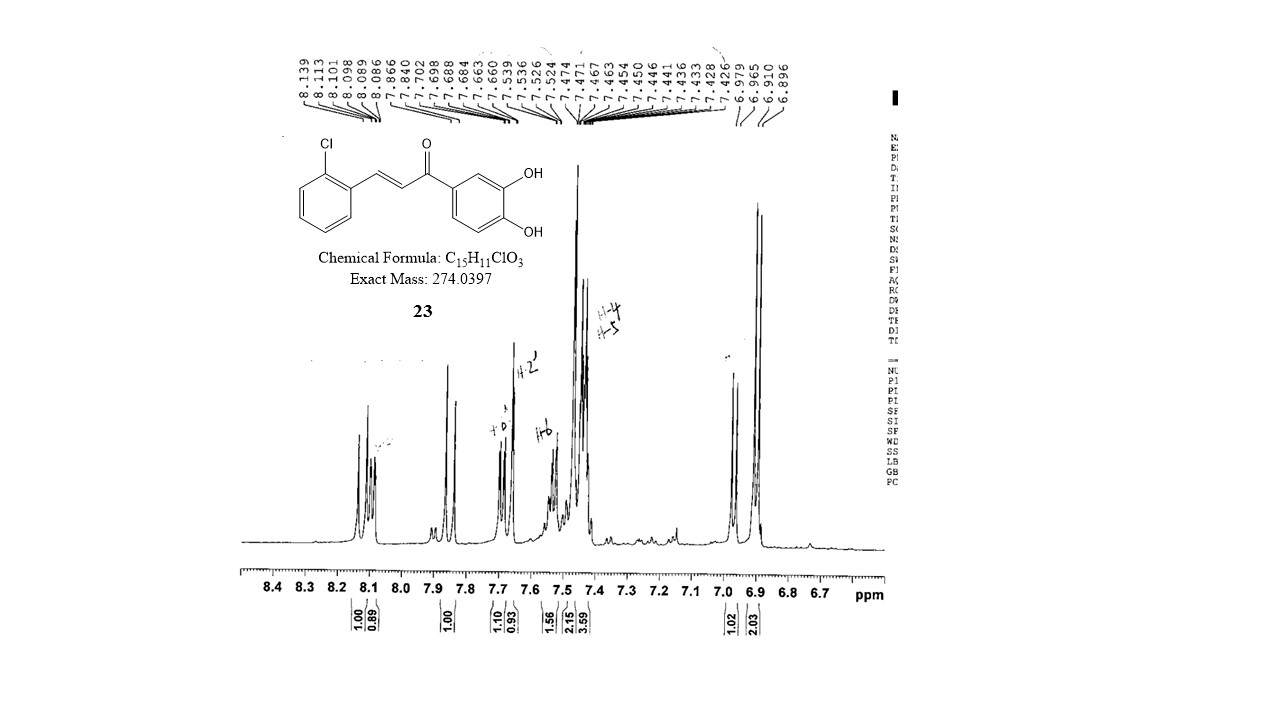

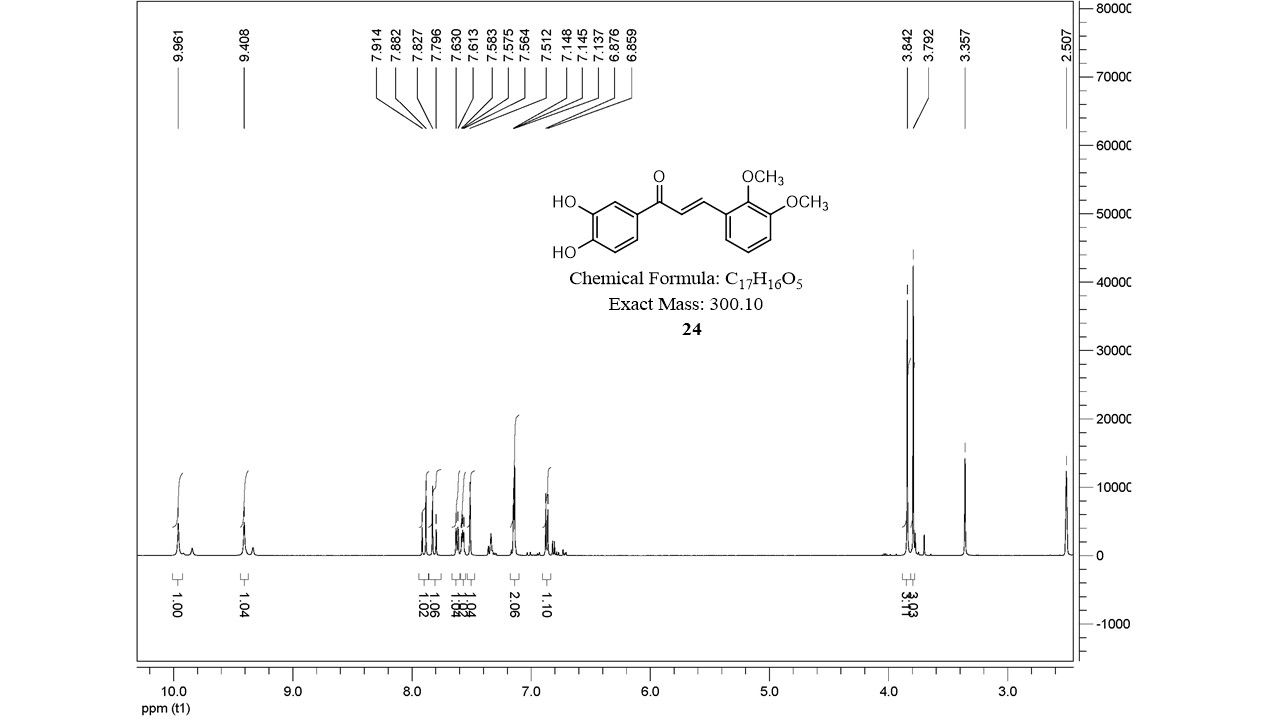


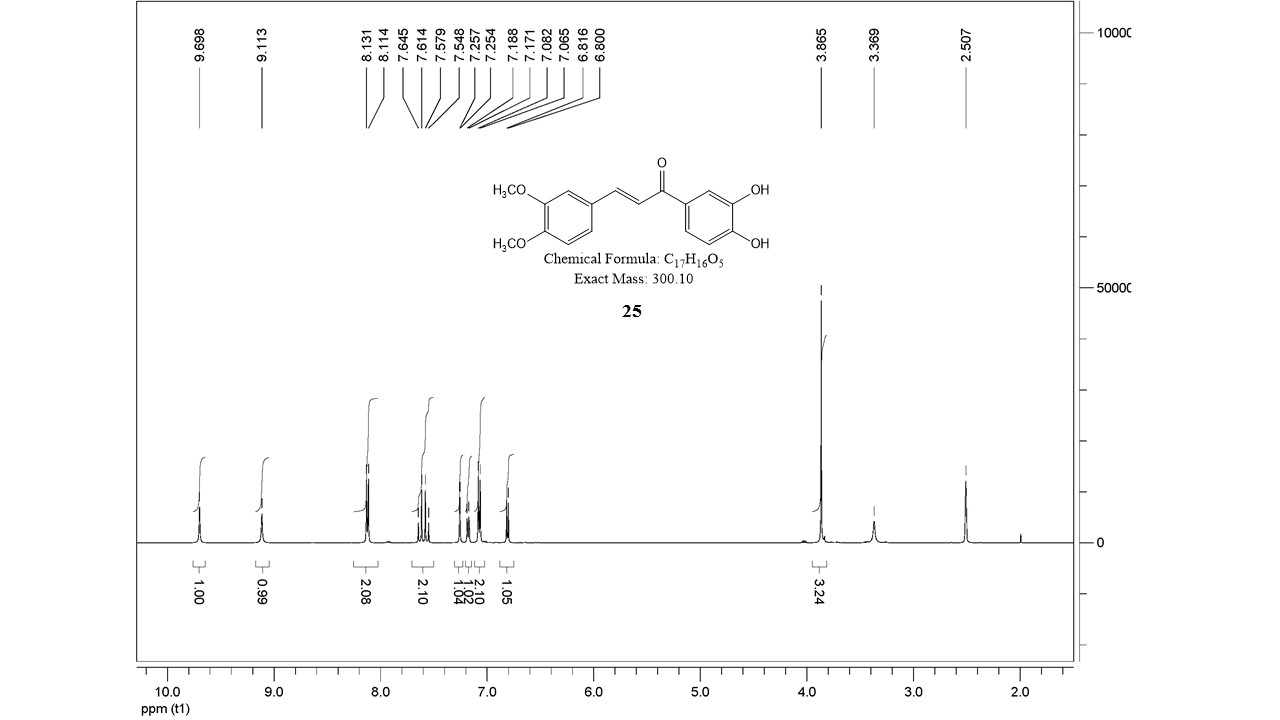

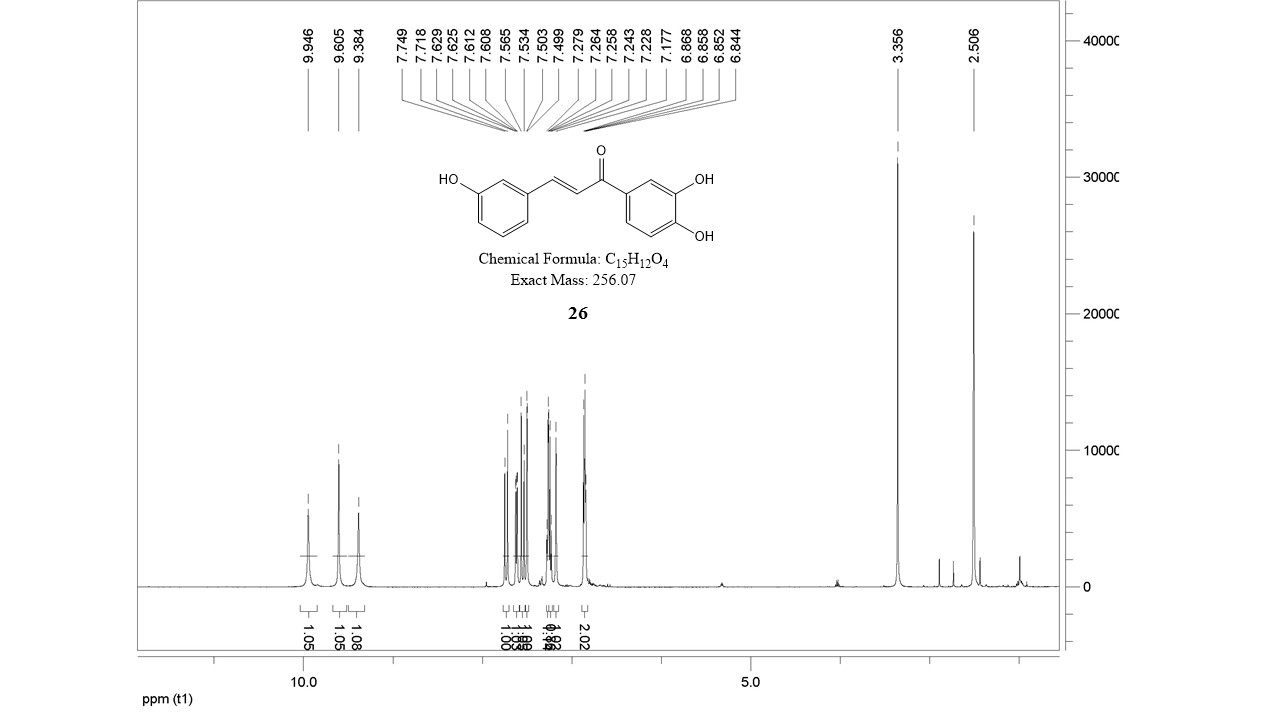


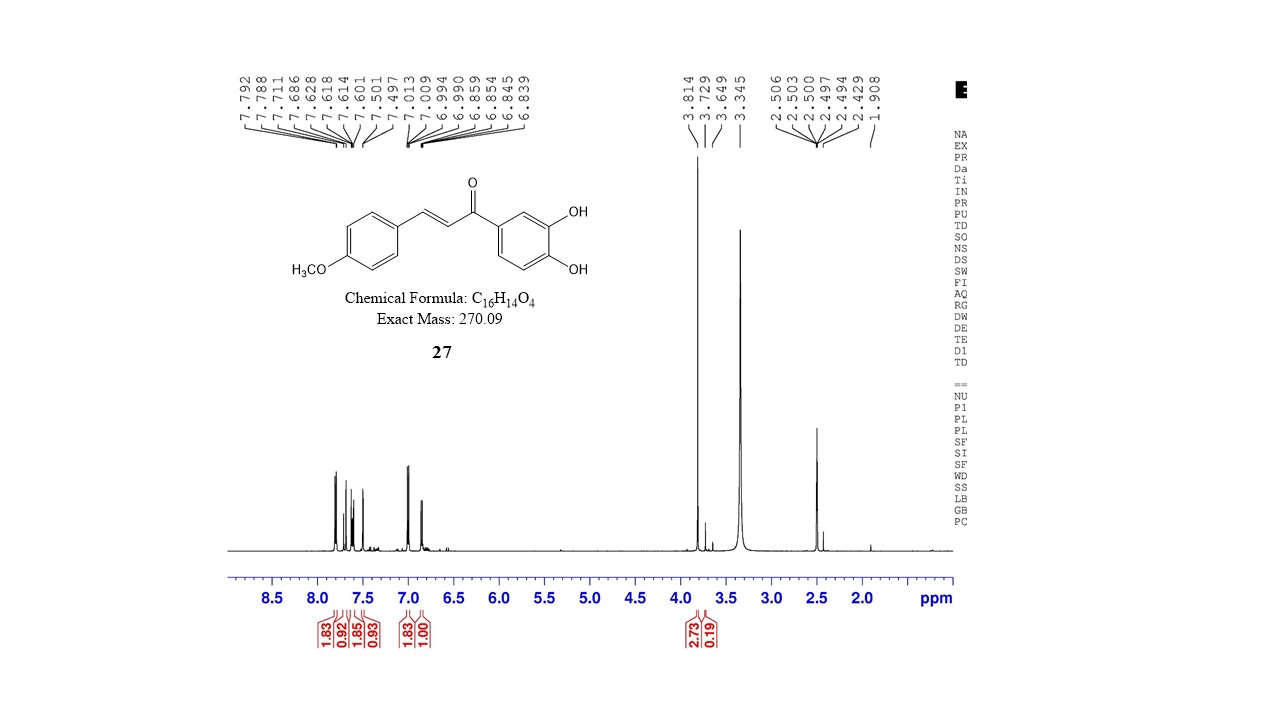

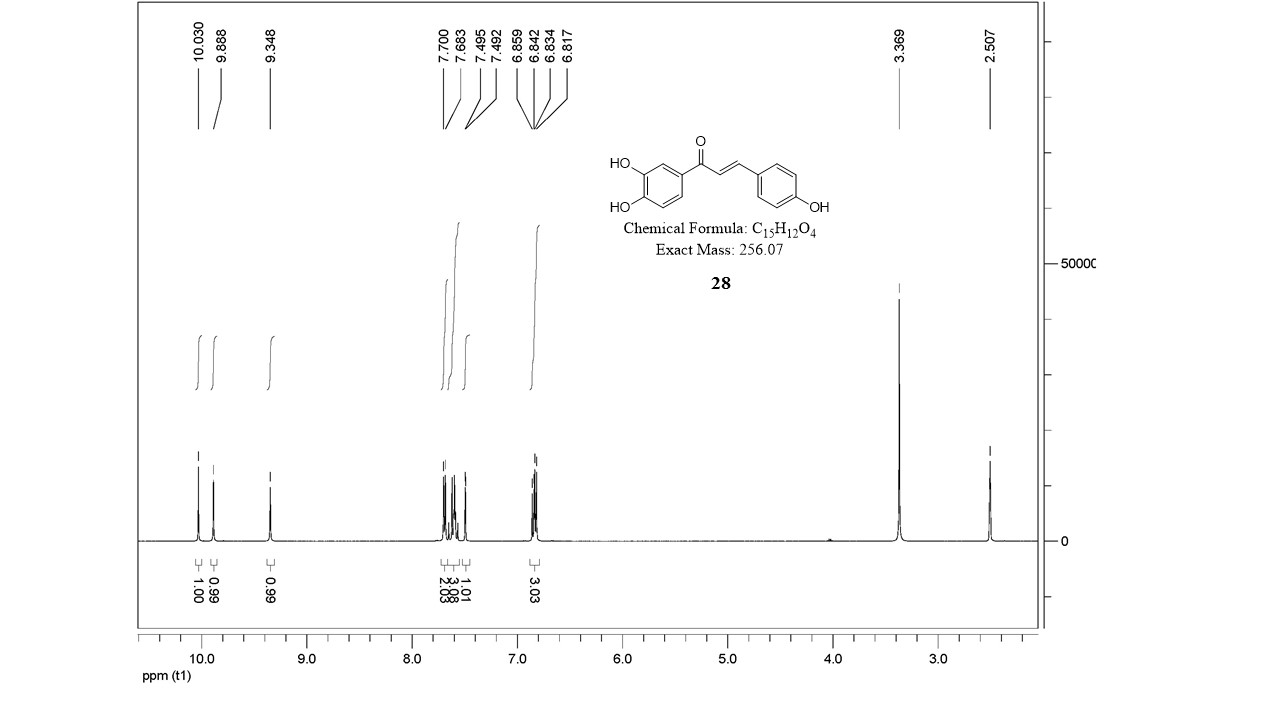


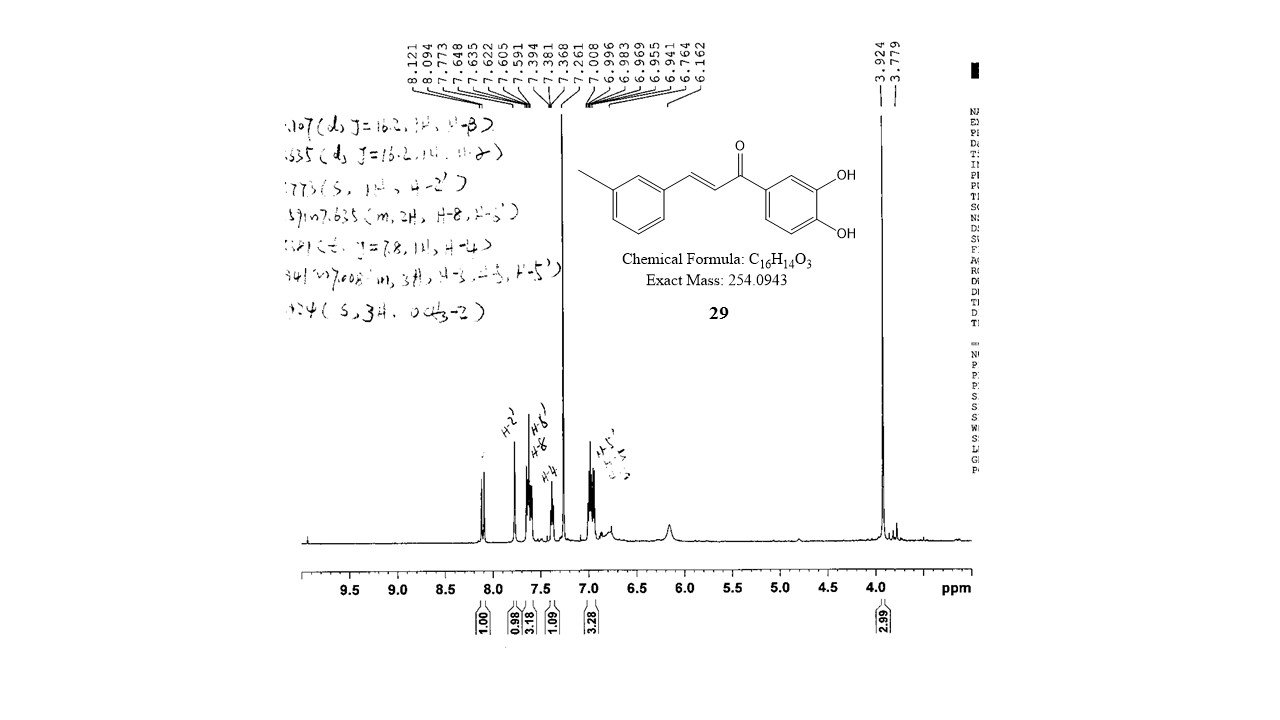

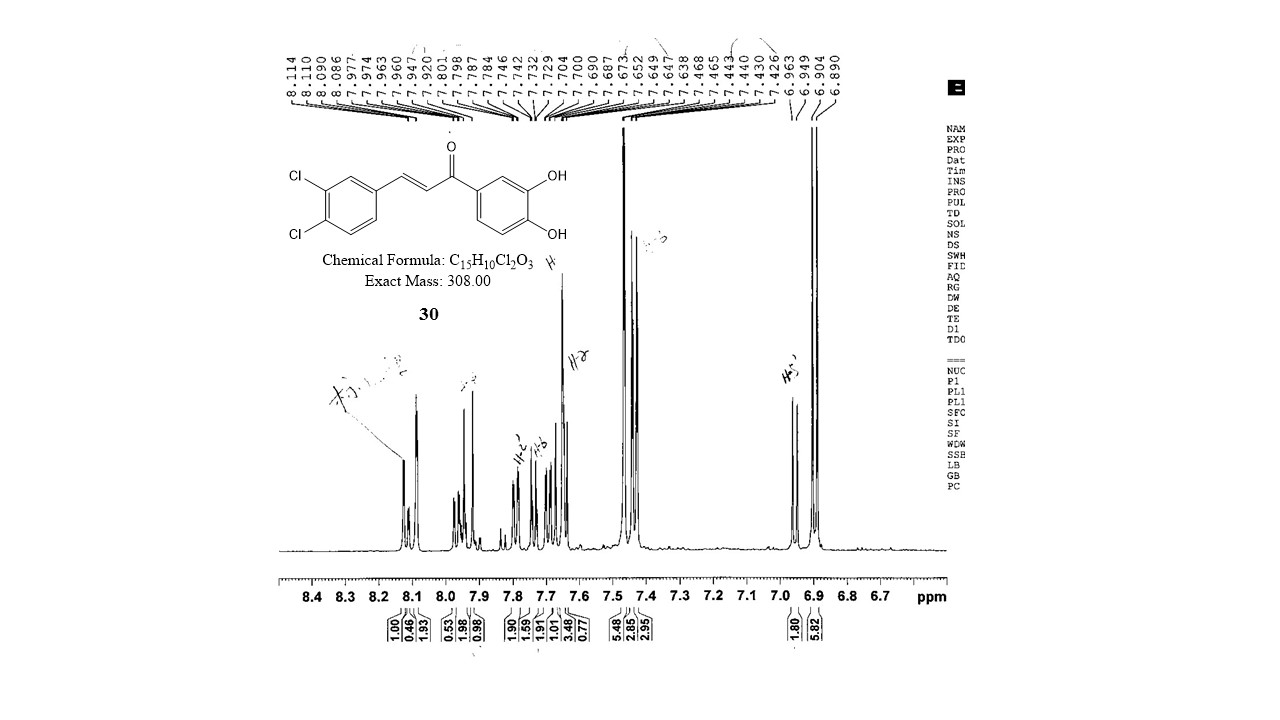


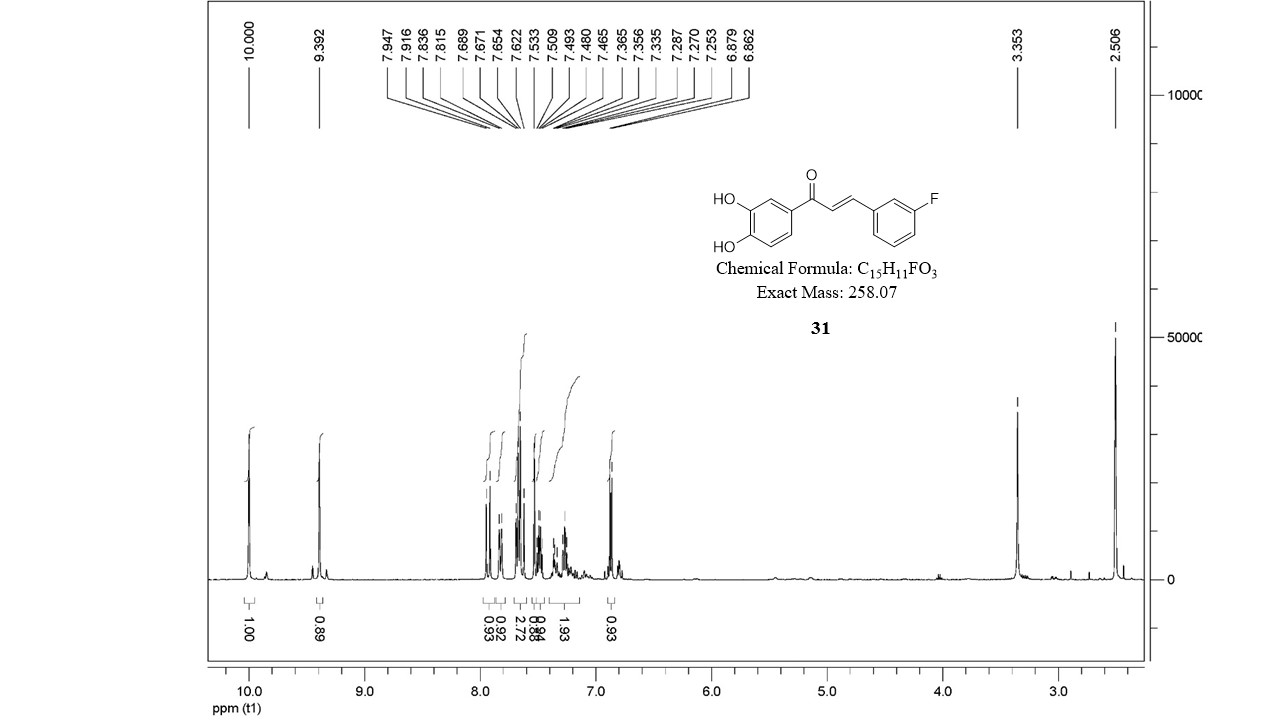


**Supplementary Figure S3.** The original ^1^H NMR spectra of compounds **1**-**31**.

**5. The cytotoxicity of active compounds**

Mouse peritoneal macrophages were seeded in 96-well plates at a concentration of 6000 cells/well and allowed to attach overnight. Macrophages were then exposure to 10 μM compound **20**, **26** and **31** in triplicate for 24 h under 5% CO_2_ at 37 ^o^C. The MTT solution (5 mg/mL in PBS) was added and the culture was incubated for an additional 4 h and absorbance was measured by using the spectraMax M5 microplate reader (MolecularDevices, Sunnyvale, CA) at 490 nm.

**Supplementary Figure S4.**  MTT assay for active compounds in macrophages

**6. Xanthohumol directly binding to MD2**

The binding affinity of xanthohumol with rhMD2 protein was determined using a ProteOn XPR36 Protein Interaction Array system (Bio-Rad Laboratories, Hercules, CA) with an HTE sensor chip (ProteOn™, #176-5033). Briefly, protein was loaded to the sensors activated with 10 mM NiSO_4_. The xanthohumol samples (at 6.25, 12.5, 25, 50, or 100 μM) were prepared with running buffer (PBS, 0.1% SDS, 5% DMSO). Sensor and sample plates were placed on the instrument, then the xanthohumol samples were captured in flow cell one, leaving the second flow cell as a blank. Five concentrations were injected simultaneously at a flow rate of 30 μM/min for a 120 s association phase, followed by a 120 s dissociation phase at 25 °C. The final graphs were obtained by subtracting blank sensor grams from the duplex or quadruplex sensorgrams. Data were analyzed with ProteOn manager software. KD was calculated by global fitting of the kinetic data from various concentrations of xanthohumol using a 1:1 Langmuir binding model.


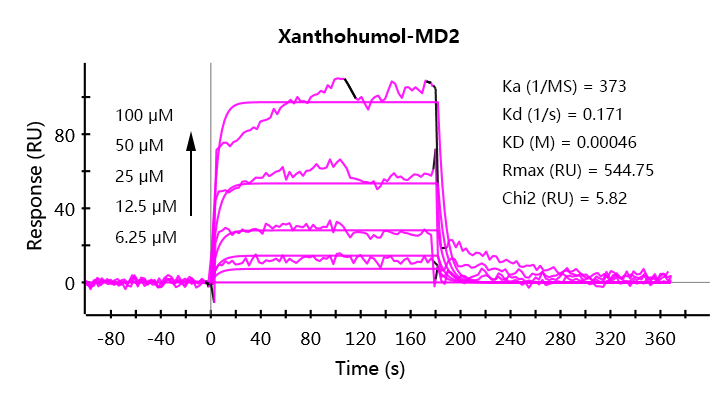


**Supplementary Figure S5.** Binding affinity of xanthohumol with rhMD2 as determined by SPR assay.

**7. The effects of compound 20 on the kinases activity of TLR4 downstream**

Kinase inhibitory activities were determined using a microfluidic assay (Caliper Mobility Shift Assay) that monitors the separation of a phosphorylated product from its substrate. Briefly, the enzyme, substrate, ATP, and compound were mixed in a 384-well assay plate before phosphorylation reaction. After 1 h incubation, EDTA was added to stop the reaction. The plate was read on an EZ Reader II (Caliper Life Sciences, MA). The percentage conversion of the substrate into the phosphorylated product was generated automatically, and the percentage inhibition was calculated relative to the blank wells, which contained no enzyme and 2.5% (v/v) DMSO, and the total wells, which containing all reagents and 2.5% (v/v) DMSO. The recombinant kinases, including ERK1/2, IRAK1, and IKK-beta, were purchased from Carna (Chuo-ku, Kobe, Japan). Compounds were tested in 3 duplicate concentrations (10, 1, and 0.1μM).


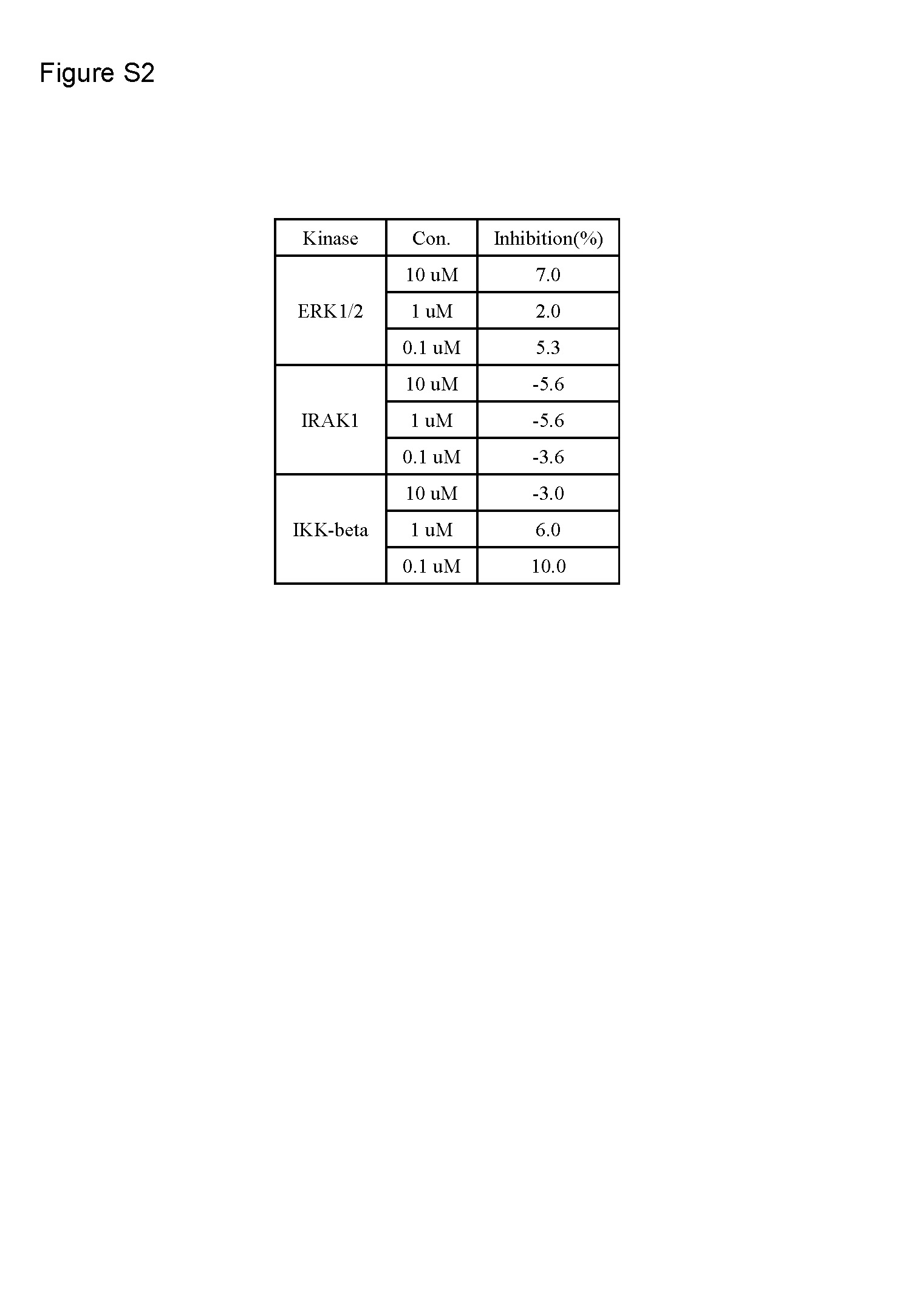


**Supplementary Figure S6.** Effects of compound **20** on kinase activity of TLR4 downstream, determined by Caliper Mobility Shift Assay.

**8. Compound 20 inhibits the inflammatory genes expression in Beas-2B cells**

Beas-2B cells were pretreatment with the vehicle control (DMSO) or compound **20** (10 μM) for 30 min followed by incubation with LPS (1 μg/mL) for 12 h. Total RNA was isolated from cells using Trizol-reagent and quantified by UV absorption at 260 and 280 nm. Both reverse transcription and quantitative PCR were performed using a two-step M-MLV Platinum SYBR Green qPCR SuperMix-UDG kit. An eppendorf Mastercycler ep realplex detection system (Eppendorf, Hamburg, Germany) was used for q-PCR analysis. The primers of genes, including TNF-α, IL-6, IL-1β, COX-2, and β-actin, were synthesized by Invitrogen. The amount of each gene was determined and normalized by the amount of β-actin.


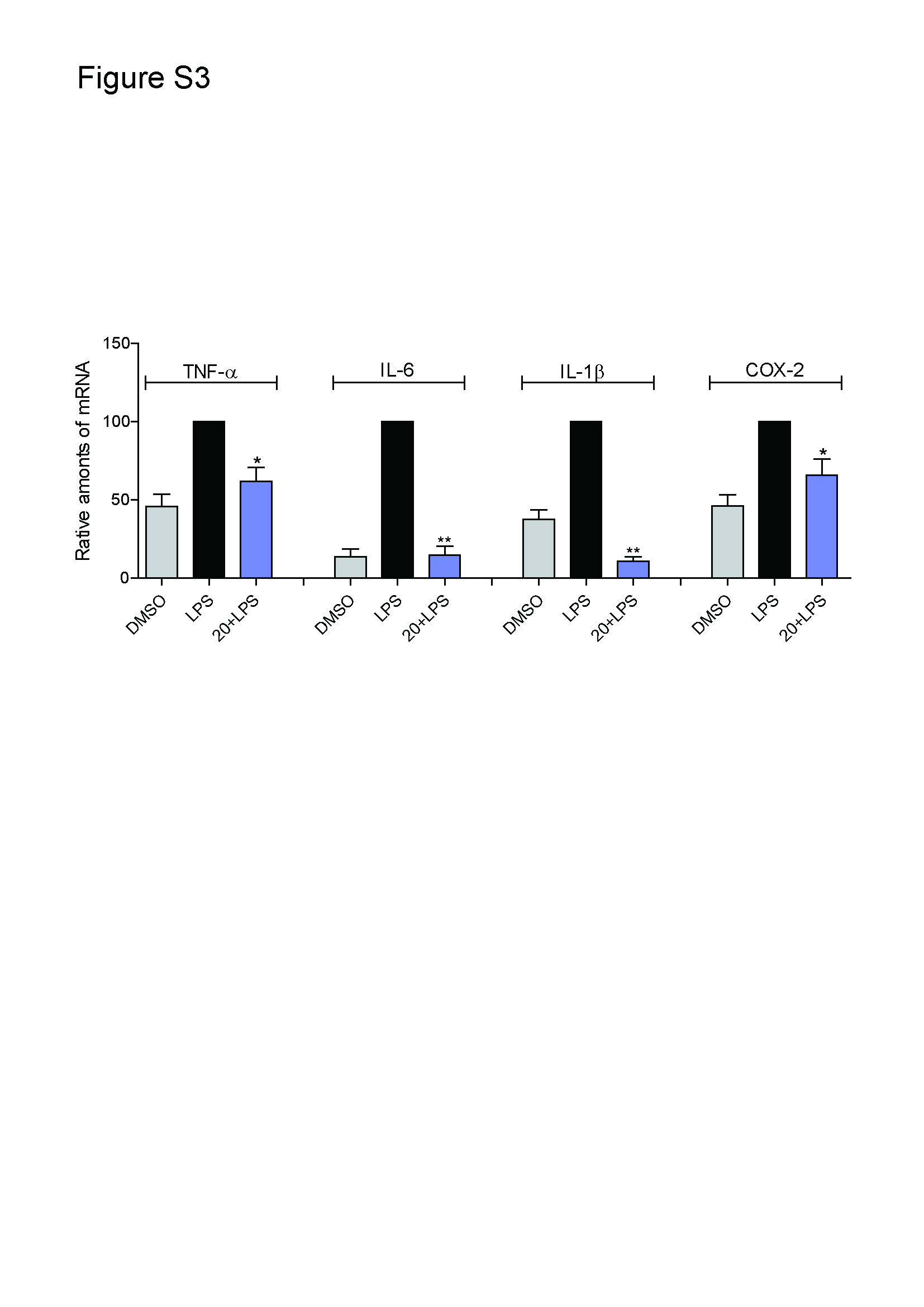


**Supplementary Figure S7.** Compound **20** inhibits the LPS-induced expression of inflammatory genes in Beas-2B.

**9. The cropping lines of gels/blots**

Selected data were shown in our manuscript and the PVDF membranes for Western Blot were cut into strips to minimize the amount of antibodies that are necessary for analysis. Cropped gels/blots are presented here.

**
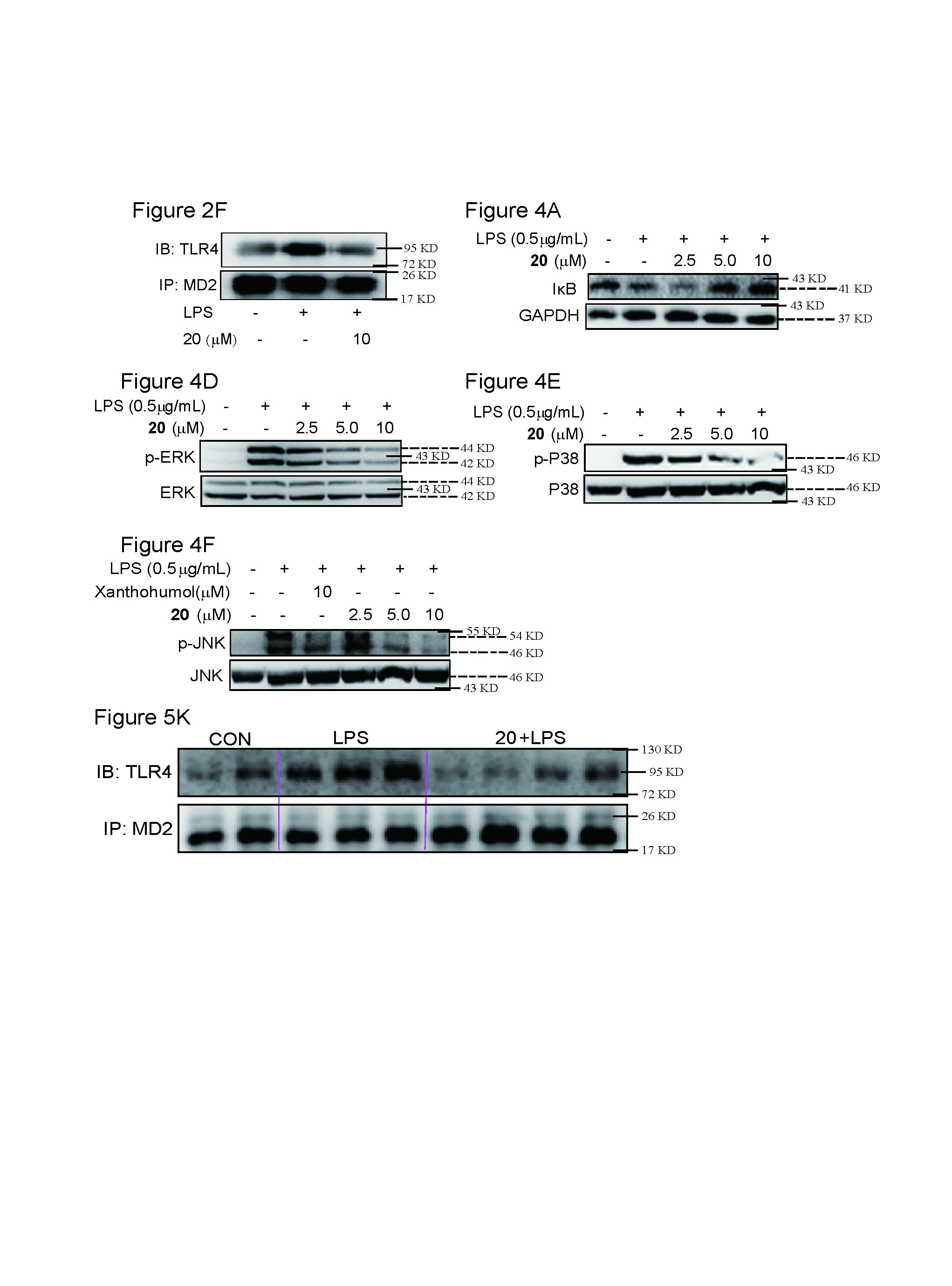
**

**Supplementary Figure S8.** The cropping lines of gels/blots.

**Supplementary References**

1 Sogawa, S. *et al.* 3,4-Dihydroxychalcones as potent 5-lipoxygenase and cyclooxygenase inhibitors. *J Med Chem* **36**, 3904-3909, doi:10.1021/jm00076a019 (1993).

2 Sharma, A. K., Yadav, A. K. & Prakash, L. Synthesis of some new pyrimidine-bases as precursors of potential anticancer nucleosides. *Indian J Chem B* **34**, 740-742 (1995).

3 Rao, Y. K., Fang, S.-H. & Tzeng, Y.-M. Synthesis and biological evaluation of 3 ',4 ',5 '-trimethoxychalcone analogues as inhibitors of nitric oxide production and tumor cell proliferation. *Bioorgan Med Chem* **17**, 7909-7914, doi:10.1016/j.bmc.2009.10.022 (2009).

4 Bhat, B. A. *et al.* Synthesis and biological evaluation of chalcones and their derived pyrazoles as potential cytotoxic agents. *Bioorgan Med Chem Lett* **15**, 3177-3180, doi:10.1016/j.bmcl.2005.03.121 (2005).

5 Lopez, S. N. *et al.* In vitro antifungal evaluation and structure-activity relationships of a new series of chalcone derivatives and synthetic analogues, with inhibitory properties against polymers of the fungal cell wall. *Bioorgan Med Chem* **9**, 1999-2013, doi:10.1016/s0968-0896(01)00116-x (2001).

6 Rostom, S. A. F., Badr, M. H., Abd El Razik, H. A., Ashour, H. M. A. & Wahab, A. E. A. Synthesis of some pyrazolines and pyrimidines derived from polymethoxy chalcones as anticancer and antimicrobial agents. *Arch Pharm* **344**, 572-587, doi:10.1002/ardp.201100077 (2011).
